# Integrated Transcriptomic and Metabolomic Analysis Reveals Molecular Responses Associated with Foliar Selenium Treatment Under Cold Stress in Rapeseed (*Brassica napus* L.) Seedlings

**DOI:** 10.3390/ijms27167424

**Published:** 2026-08-19

**Authors:** Long Wei, Ji Wang, Qian Cui, Yitong Xia, Songhui Wang, Mengjia Zhou

**Affiliations:** 1 School of Geography and Planning, Chizhou University, Chizhou 247000, China; 2 Research Center for Agricultural Ecological Resources and Environment, Chizhou University, Chizhou 247000, China; 3 State Key Laboratory of Crop Genetics & Germplasm Enhancement and Utilization, Nanjing Agricultural University, Nanjing 210095, China

**Keywords:** *Brassica napus*, selenium, cold stress, transcriptome, metabolome

## Abstract

Rapeseed (*Brassica napus* L.) is an important oil crop, but seedling growth is often restricted by low temperatures in late autumn and winter. Selenium (Se) may modulate plant stress responses, but its dose-dependent effects and associated molecular responses in rapeseed under cold stress remain unclear. Seedlings were exposed to 4 °C for 3 d and received foliar applications of 10, 20, or 40 mg L^−1^ Na_2_SeO_3_. Based on physiological responses, the 20 mg L^−1^ treatment was selected for integrated transcriptomic and metabolomic analyses alongside the normal-temperature control and the cold-stress group without Se. Cold stress reduced whole-seedling fresh weight by approximately 40% and increased O_2_^−^ production, H_2_O_2_ and malondialdehyde (MDA) contents, and superoxide dismutase (SOD), peroxidase (POD), and catalase (CAT) activities. Relative to the cold-stress group without Se, the 20 mg L^−1^ treatment showed partial restoration of fresh weight and lower oxidative-stress indicators and antioxidant enzyme activities. Integrated analyses highlighted cysteine and methionine metabolism and phenylpropanoid biosynthesis. Treatment-associated changes involved sulfur-related and methyl-cycle genes, a metabolite feature tentatively assigned to 3-sulfopyruvic acid, and a feature putatively annotated as coniferaldehyde. These findings provide a multi-omics basis for understanding rapeseed responses to foliar-applied selenite during short-term cold stress.

## 1. Introduction

Rapeseed (*Brassica napus* L.) is one of the main oil crops in the world. It supplies edible oil for people and meal for livestock [1]. Rapeseed is a cool-season crop and is widely grown in temperate and subtropical regions. In these areas, seedlings raised in late autumn and winter often meet low temperatures [2]. Cold stress is therefore a major problem in rapeseed production. It slows seed germination, weakens seedling growth, and lowers the final yield and oil quality [3]. Reducing cold damage at the seedling stage is thus important for stable production.

Cold stress can lead to the accumulation of reactive oxygen species (ROS), such as the superoxide anion (O_2_^−^) and hydrogen peroxide (H_2_O_2_) [4]. Excess ROS damage membrane lipids, proteins, and nucleic acids. This is shown by a rise in malondialdehyde (MDA) and by the loss of normal cell function [5]. Plants limit this damage through antioxidant enzymes, including superoxide dismutase (SOD), peroxidase (POD), and catalase (CAT), together with non-enzymatic antioxidants such as phenolic and flavonoid compounds [6,7]. Among these enzymes, class III peroxidases are particularly relevant because they participate in ROS homeostasis and also contribute to phenolic oxidation and lignin-related cell-wall processes. Recent functional studies have further shown that individual class III peroxidases can regulate ROS homeostasis under stress and that cold-responsive peroxidases can contribute to lignin accumulation and low-temperature tolerance [8,9]. Peroxidases may therefore link oxidative responses with phenylpropanoid-related processes during cold stress.

Research on cold tolerance in rapeseed has progressed from physiological characterization to genetic and multi-omics analyses. Integrated transcriptomic and metabolomic analysis of low-temperature germination in *Brassica napus* identified coordinated changes in carbohydrate and amino acid metabolism, including pathways related to glutathione metabolism and starch and sucrose metabolism [10]. Recent transcriptomic studies have further characterized genotype-dependent cold responses in rapeseed seedlings [11]. BSA-seq, GWAS, and transcriptomic analyses have also identified candidate loci and genes associated with seedling cold tolerance and temperature-dependent germination [2,3]. In addition, integrated omics analysis has shown that treatment with an exogenous regulator is associated with changes in pathways related to amino acid and carbohydrate metabolism, phenylpropanoid biosynthesis, and other secondary metabolic processes during cold stress in rapeseed [12]. Together, these studies indicate that cold adaptation in rapeseed involves coordinated changes in redox regulation, gene expression, and primary and secondary metabolism.

Among the exogenous treatments investigated for improving abiotic stress tolerance, selenium (Se) has received increasing attention. Although Se is not essential for higher plants, it can promote plant growth and modulate responses to abiotic stress when supplied at appropriate doses [13]. Sodium selenite (Na_2_SeO_3_) is a commonly used inorganic Se source in agriculture, and foliar application is a practical method for its delivery. The effect of Se depends strongly on the dose. A suitable concentration relieves stress, while higher concentrations may exacerbate oxidative damage [14]. However, the effective foliar Na_2_SeO_3_ concentration for cold-stressed rapeseed remains poorly defined.

At the metabolic level, Se is closely linked to sulfur metabolism, although the uptake and early metabolism of inorganic Se vary with chemical form. Selenate is commonly transported via sulfate transporters and subsequently enters the sulfur assimilation pathway, whereas selenite follows distinct uptake and initial conversion routes [15]. Once incorporated into cellular metabolism, Se can interact with cysteine and methionine metabolism and with the S-adenosylmethionine cycle [15,16]. Phenylpropanoid metabolism represents another relevant stress-response pathway because it supplies phenolic compounds and monolignol precursors and is closely linked to redox regulation and cell-wall metabolism [17]. Class III peroxidases further connect these processes through their involvement in H_2_O_2_-dependent reactions and lignin-related metabolism [8,9]. Whether foliar-applied selenite under cold conditions is accompanied by coordinated changes in sulfur-related metabolism, phenylpropanoid metabolism, and peroxidase responses in rapeseed remains unclear.

Integrated transcriptomic and metabolomic analysis provides an effective approach for examining coordinated changes in gene expression and metabolite abundance at the pathway level [18]. This strategy has been increasingly used to investigate plant responses to environmental stress and exogenous regulators [12,18,19]. However, integrated omics studies of foliar sodium selenite treatment during cold stress in rapeseed remain scarce. The associated genes, metabolites, and pathways have not been characterized systematically.

Accordingly, the novelty of the present study lies in combining a foliar Na_2_SeO_3_ concentration-response assessment with physiological measurements and integrated transcriptomic and metabolomic analyses under cold conditions. We hypothesized that an appropriate foliar Na_2_SeO_3_ treatment would be associated with measurable differences in whole-seedling fresh weight, oxidative status, and selected metabolic pathways under the applied cold conditions. Rapeseed seedlings exposed to 4 °C were therefore treated with several Na_2_SeO_3_ concentrations. Whole-seedling fresh weight, antioxidant enzyme activities, and oxidative-stress indicators were measured first to find the most effective concentration within the tested range. Samples from the selected treatment, the untreated cold-stress group, and the normal-temperature control were then subjected to integrated transcriptomic and metabolomic analyses. The study aimed to (1) identify the Na_2_SeO_3_ concentration associated with the most favorable physiological response within the tested range; (2) identify differentially expressed genes and differentially accumulated metabolites associated with Se treatment under cold stress; and (3) identify pathways jointly highlighted by the transcriptomic and metabolomic datasets. The results provide a framework for interpreting the physiological and molecular responses associated with sodium selenite treatment under cold conditions.

## 2. Results

### 2.1. Effects of Foliar Na_2_SeO_3_ on Whole-Seedling Fresh Weight Under Cold Conditions

**Figure 1:**
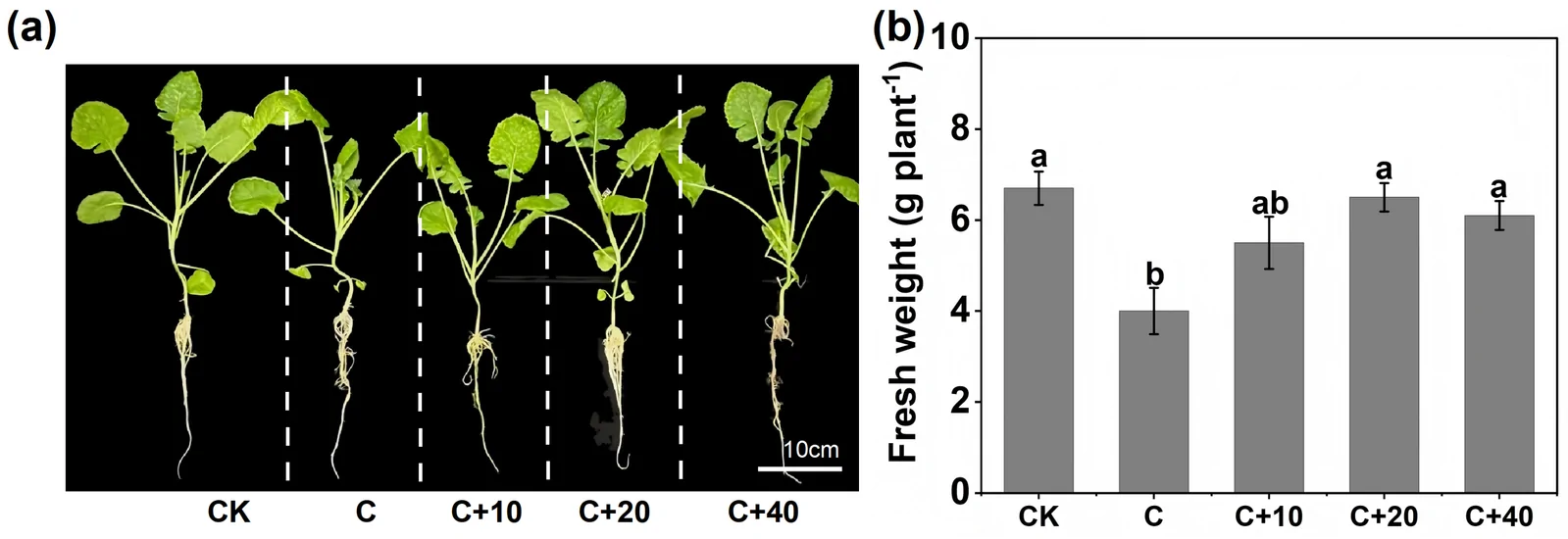
Effects of foliar Na_2_SeO_3_ on the visible phenotype and whole-seedling fresh weight of rapeseed under cold stress. (**a**) Representative phenotypes of rapeseed seedlings under the five treatments after 3 d of cold treatment. CK, control kept under normal growth conditions without selenium or cold stress; C, cold stress at 4 °C without selenium; C+10, C+20 and C+40, cold stress at 4 °C with a foliar spray of sodium selenite (Na_2_SeO_3_) at 10, 20 and 40 mg L^−1^, respectively. Scale bar = 10 cm. (**b**) Fresh weight per plant. Data are presented as the mean±SD of three independent box-level biological replicates (*n* = 3). Different lowercase letters indicate significant differences among treatment means according to Duncan’s multiple range test following one-way ANOVA. Means sharing at least one letter are not significantly different, whereas means with no letter in common differ significantly at *p* < 0.05.

Cold stress clearly inhibited the growth of rapeseed. The seedlings under cold stress without Se (C) were smaller than the normal-temperature control (CK) seedlings, and their fresh weight decreased by about 40% compared with CK (Figure 1). Relative to the C group, whole-seedling fresh weight was 38%, 63%, and 53% higher in the C+10, C+20, and C+40 groups, respectively. Fresh weight in the C+20 and C+40 groups did not differ significantly from that in CK, and the greatest increase relative to C was observed in the C+20 group (Figure 1b). Thus, foliar Na_2_SeO_3_ attenuated the cold-associated reduction in whole-seedling fresh weight, with the greatest response observed at 20 mg L^−1^ among the concentrations tested.

### 2.2. Effects of Se Treatment on Antioxidant Enzyme Activities and Oxidative Stress Markers Under Cold Stress

**Figure 2:**
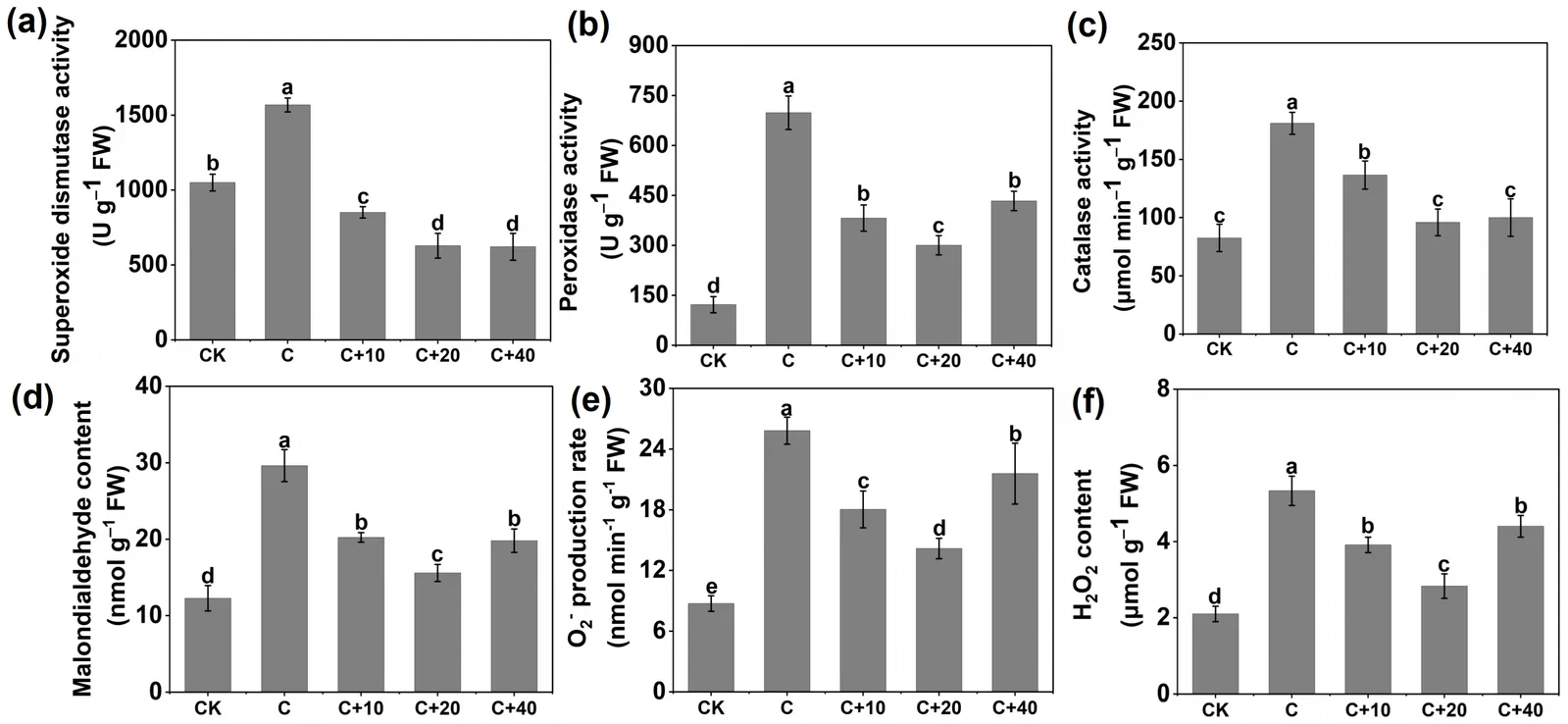
Effect of selenium on antioxidant enzyme activities and reactive oxygen species accumulation in rapeseed seedlings under cold stress. (**a**) Superoxide dismutase (SOD) activity, (**b**) peroxidase (POD) activity, (**c**) catalase (CAT) activity, (**d**) malondialdehyde (MDA) content, (**e**) superoxide anion (O_2_^−^) production rate, and (**f**) hydrogen peroxide (H_2_O_2_) content. CK, well-watered control; C, 4 °C cold stress; C+10, C+20 and C+40, cold stress supplemented with 10, 20 and 40 mg L^−1^ Na_2_SeO_3_, respectively. Data are presented as the mean ± SD of three independent box-level biological replicates (*n* = 3). Different lowercase letters indicate significant differences among treatment means according to Duncan’s multiple range test following one-way ANOVA. Means sharing at least one letter are not significantly different, whereas means with no letter in common differ significantly at *p* < 0.05. All biochemical assays were performed using whole-seedling tissue collected at the end of the 3-d treatment.

The activities of antioxidant enzymes and the levels of reactive oxygen species (ROS) in rapeseed seedlings under different treatments are shown in Figure 2. Compared to the CK treatment, the C treatment significantly increased the activities of superoxide dismutase (SOD), peroxidase (POD) and catalase (CAT) by 50.0%, 483.3%, and 122.0% (Figure 2a–c), as well as the malondialdehyde (MDA) content, superoxide anion (O_2_^−^) production rate, and hydrogen peroxide (H_2_O_2_) content by 145.8%, 183.3%, and 152.4% (Figure 2d–f), respectively.

Notably, relative to the C treatment, the C+20 treatment was associated with decreases in MDA content, O_2_^−^ production rate, and H_2_O_2_ content of 47.1%, 45.1%, and 46.2%, respectively (Figure 2d–f), and these three values were the lowest among all treatments and close to the CK level. In contrast, the C+40 treatment showed higher O_2_^−^ production rate and H_2_O_2_ content than C+20 (Figure 2e–f). The C+20 treatment also showed lower SOD, POD, and CAT activities than the C treatment (Figure 2a–c), with the SOD activity even falling below the CK level. The SOD, POD, and CAT activities were also lower in the C+10 and C+40 treatments than in the C treatment. (Figure 2a–c), but the decreases were smaller than those in the C+20 treatment. At the 3-d endpoint, seedlings in the C+20 treatment had the highest whole-seedling fresh weight and the lowest O_2_^−^ production rate and H_2_O_2_ and MDA contents among the cold-treated groups. Therefore, this treatment was selected for transcriptomic and metabolomic analyses and is hereafter designated CSe.

### 2.3. Transcriptome Profiling and Differentially Expressed Genes (DEGs) of Rapeseed Seedlings Under Cold Stress with Se Supply

The nine RNA-seq libraries yielded 63.01 Gb of clean bases in total, with 6.71–8.08 Gb obtained per library. The Q30 values ranged from 97.70% to 99.00%, the GC content ranged from 46.52% to 47.42%, and the mapping rates ranged from 94.38% to 99.16% (Table S1). These results indicated that the RNA-seq data were of sufficient quality for subsequent analyses.

**Figure 3:**
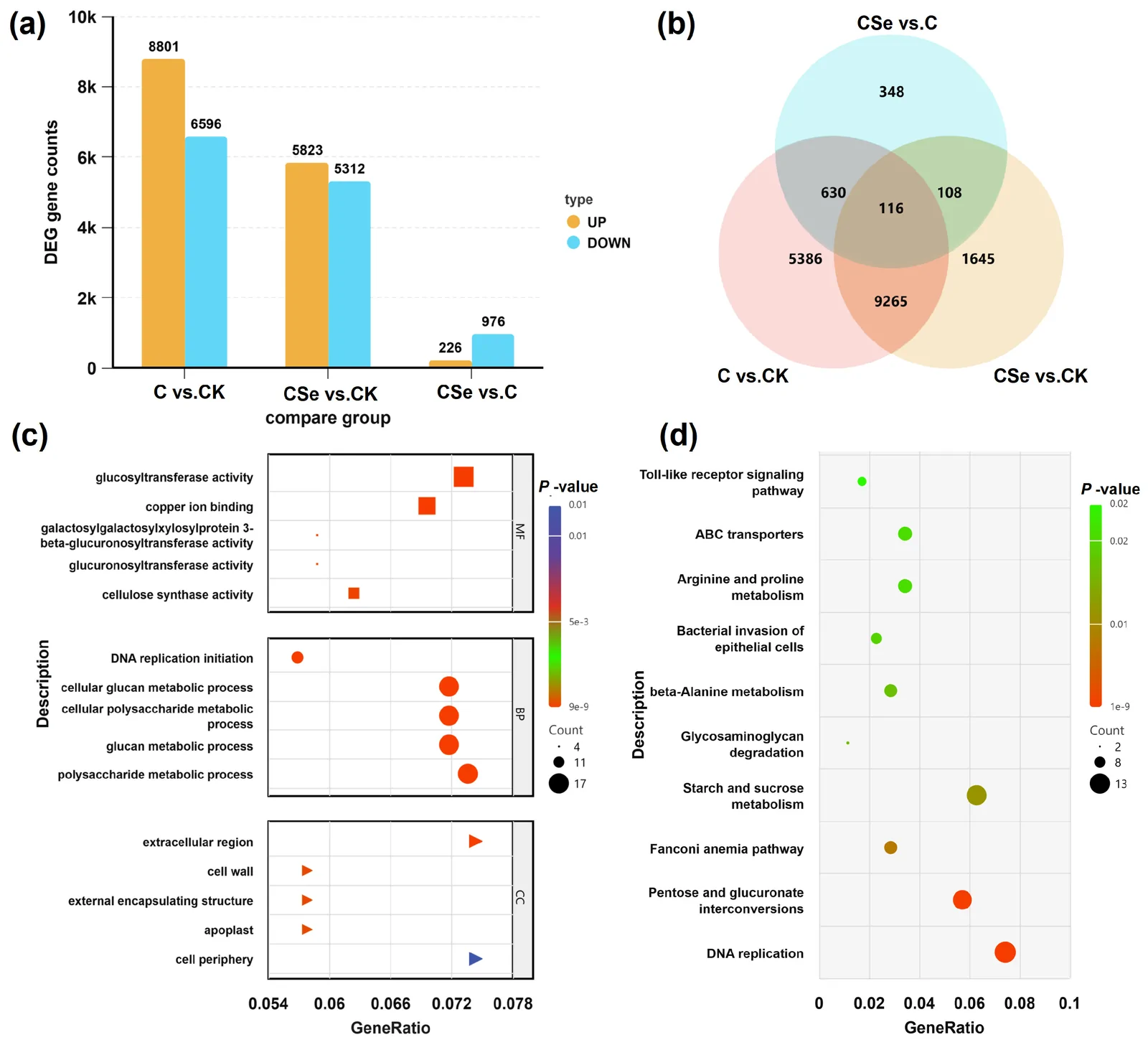
Transcriptomic responses of rapeseed to selenium under cold stress. (**a**) Numbers of up- and down-regulated differentially expressed genes (DEGs) in the C vs. CK, CSe vs. CK and CSe vs. C comparisons. (**b**) Venn diagram showing the shared and unique DEGs among the three comparisons. (**c**) Gene Ontology (GO) enrichment of the DEGs in the CSe vs. C comparison. Squares, circles, and right-pointing triangles denote terms in the molecular function (MF), biological process (BP), and cellular component (CC) categories, respectively. (**d**) Kyoto Encyclopedia of Genes and Genomes (KEGG) pathway enrichment of the DEGs in the CSe vs. C comparison. In (**c**,**d**), the *x*-axis represents the GeneRatio, symbol size indicates the number of DEGs, and symbol color represents the BH-adjusted *p*-value. CK, well-watered control; C, 4 °C cold stress; CSe, cold stress supplemented with 20 mg L^−1^ Na_2_SeO_3_.

Transcriptome sequencing was performed on whole-seedling samples from the CK, C, and CSe groups, and DEGs were identified in three pairwise comparisons. In the C vs. CK comparison, a total of 15,397 DEGs were identified, comprising 8801 up-regulated and 6596 down-regulated genes (Figure 3a). For the CSe vs. CK comparison group, 11,135 DEGs were identified, including 5823 up-regulated and 5312 down-regulated genes (Figure 3a). A total of 1202 DEGs were identified in the CSe vs. C comparison, of which 226 were up-regulated and 976 were down-regulated (Figure 3a). Among the DEGs, 116 were commonly identified in the three comparison groups, and 348 DEGs were unique to the CSe vs. C comparison group (Figure 3b). The DEGs in the C vs. CK and CSe vs. CK comparisons were mainly enriched in photosynthesis-related and carbohydrate-related processes (Figures S1 and S2), consistent with broad transcriptional responses of cold-exposed seedlings under the specified light regime. Because the experimental design did not include a group treated with Se under normal temperature, the CSe vs. C comparison was used to identify changes associated with Se treatment under cold conditions. GO and KEGG over-representation analyses identified terms and pathways with Benjamini–Hochberg adjusted *p*-values below 0.05 in the CSe vs. C comparison. In the GO analysis, the DEGs were enriched in glucosyltransferase activity, copper ion binding and cellulose synthase activity (MF); polysaccharide and glucan metabolic processes (BP); and cell wall, apoplast and extracellular region (CC) (Figure 3c). In the KEGG analysis, the DEGs were enriched in starch and sucrose metabolism, pentose and glucuronate interconversions, DNA replication, ABC transporters and arginine and proline metabolism (Figure 3d). To confirm the RNA-seq data, several DEGs were analyzed by qRT-PCR. The expression trends of the selected genes were consistent between RNA-seq and qRT-PCR (Figure S3).

### 2.4. Metabolome Information and Differentially Accumulated Metabolites (DAMs) of Rapeseed Seedlings Under Cold Stress with Se Supply

**Figure 4:**
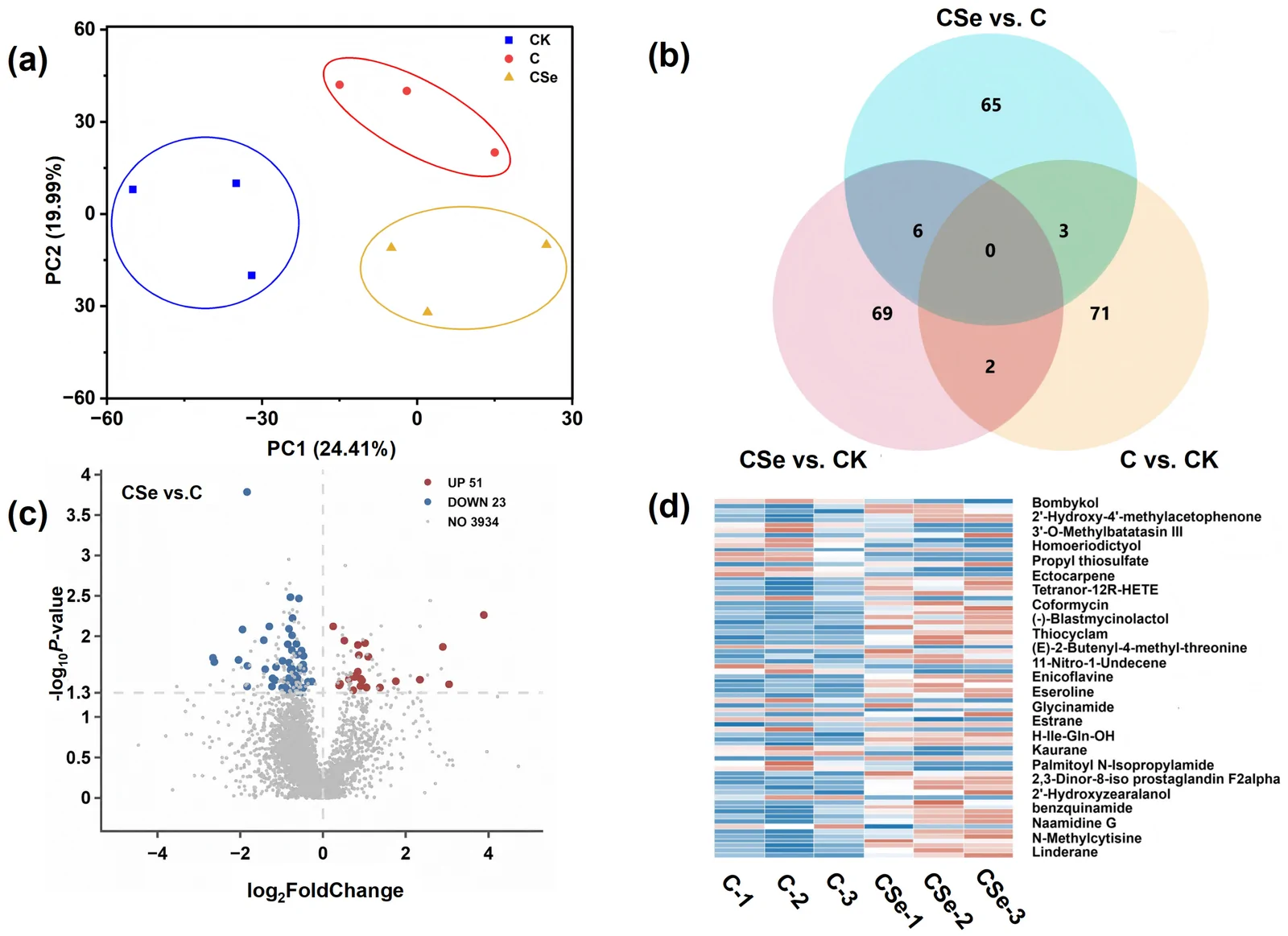
Metabolomic responses of rapeseed to selenium under cold stress. (**a**) Principal component analysis (PCA) score plot of the CK, C and CSe groups. PC1 and PC2 explained 24.41% and 19.99% of the total variance, respectively. Each point represents one biological replicate (*n* = 3) and ellipses denote the 95% confidence intervals. (**b**) Venn diagram of the differentially accumulated metabolites (DAMs) among the C vs. CK, CSe vs. CK and CSe vs. C comparisons. (**c**) Volcano plot of metabolites in the CSe vs. C comparison; red and blue dots denote candidate features with higher or lower abundance and grey dots denote metabolite features with no significant change. (**d**) Heatmap of the DAMs between the C and CSe groups (C-1–C-3 and CSe-1–CSe-3); values were row-scaled (Z-score). Hierarchical clustering was performed using Pearson correlation-based distance and complete linkage. CK, well-watered control; C, 4 °C cold stress; CSe, cold stress supplemented with 20 mg L^−1^ Na_2_SeO_3_.

Marked changes in the metabolite profiles of rapeseed seedlings were observed when Se was supplied under cold stress. Untargeted metabolomics was performed on whole-seedling samples from the same three groups (CK, C and CSe). PC1 and PC2 explained 24.41% and 19.99% of the total variance, respectively. The PCA score plot clearly separated the three groups, and the three biological replicates within each group clustered together (Figure 4a), indicating distinct treatment-associated metabolite profiles and good within-group consistency. DAMs were then screened in the three pairwise comparisons. The features meeting the combined criteria of VIP > 1, nominal *p* < 0.05, and FC ≥ 1.5 or FC ≤ 0.67 were treated as candidate DAMs for exploratory analyses. The Venn diagram showed that 71, 69, and 65 candidate DAMs were unique to the C vs. CK, CSe vs. CK, and CSe vs. C comparisons, respectively. Six candidate DAMs were shared between CSe vs. CK and CSe vs. C, three were shared between C vs. CK and CSe vs. C, and two were shared between C vs. CK and CSe vs. CK. No candidate DAM was common to all three comparisons (Figure 4b). Thus, under the applied screening criteria, the candidate DAM sets were largely comparison-specific. In the CSe vs. C comparison, 74 candidate DAMs met the applied screening criteria. Among them, 51 showed higher abundance and 23 showed lower abundance in CSe than in C (Figure 4c). Feature-level LC–MS information for the candidate DAMs in the CSe vs. C comparison is provided in Table S2. The heatmap further showed distinct abundance patterns of these candidate DAMs between the C and CSe groups (Figure 4d). In the C vs. CK comparison, the DAMs were mainly enriched in glutathione metabolism, arginine and proline metabolism, carotenoid biosynthesis and plant hormone signal transduction, reflecting the overall metabolic response to cold stress. In the CSe vs. CK comparison, selenocompound metabolism showed the strongest nominal enrichment, followed by flavonoid biosynthesis and glucosinolate biosynthesis (Figure S4). Because the experimental design did not include a group treated with Se under normal temperature, the CSe vs. C comparison was used to identify changes associated with Se treatment under cold conditions. KEGG over-representation analysis identified nominal enrichment of phenylpropanoid biosynthesis, flavonoid biosynthesis, cysteine and methionine metabolism, and one carbon pool by folate in the CSe vs. C comparison (Figure S5). These results were based on nominal *p* values and were used for exploratory pathway screening.

### 2.5. Classification of Molecular Response Patterns Across CK, C, and CSe

To distinguish cold-responsive changes from those associated with Se treatment under cold conditions, DEGs and candidate DAMs were classified according to their patterns across CK, C, and CSe (Figure S6). Among the 15,397 cold-responsive DEGs identified in C vs. CK, 630 returned to the CK range in CSe, 94 shifted toward CK but remained significantly different from CK, and 22 changed further in the cold-responsive direction. The remaining 14,651 genes showed no statistically detectable difference between C and CSe. In addition, 456 DEGs were detected in CSe vs. C but not in C vs. CK and were therefore classified as changes associated with Se treatment under cold conditions rather than reversals of cold-responsive changes (Figure S6a).

Among the 76 cold-responsive candidate DAMs, three returned to the CK range in CSe, whereas the remaining 73 showed no statistically detectable difference between C and CSe. An additional 71 candidate DAMs were detected in CSe vs. C but not in C vs. CK and were classified as Se-associated changes under cold conditions (Figure S6b). Overall, Se treatment was associated with changes in a limited subset of cold-responsive molecular features, while most cold-responsive DEGs and candidate DAMs did not differ significantly between C and CSe.

### 2.6. Integrated Transcriptomic and Metabolomic Analysis

**Figure 5:**
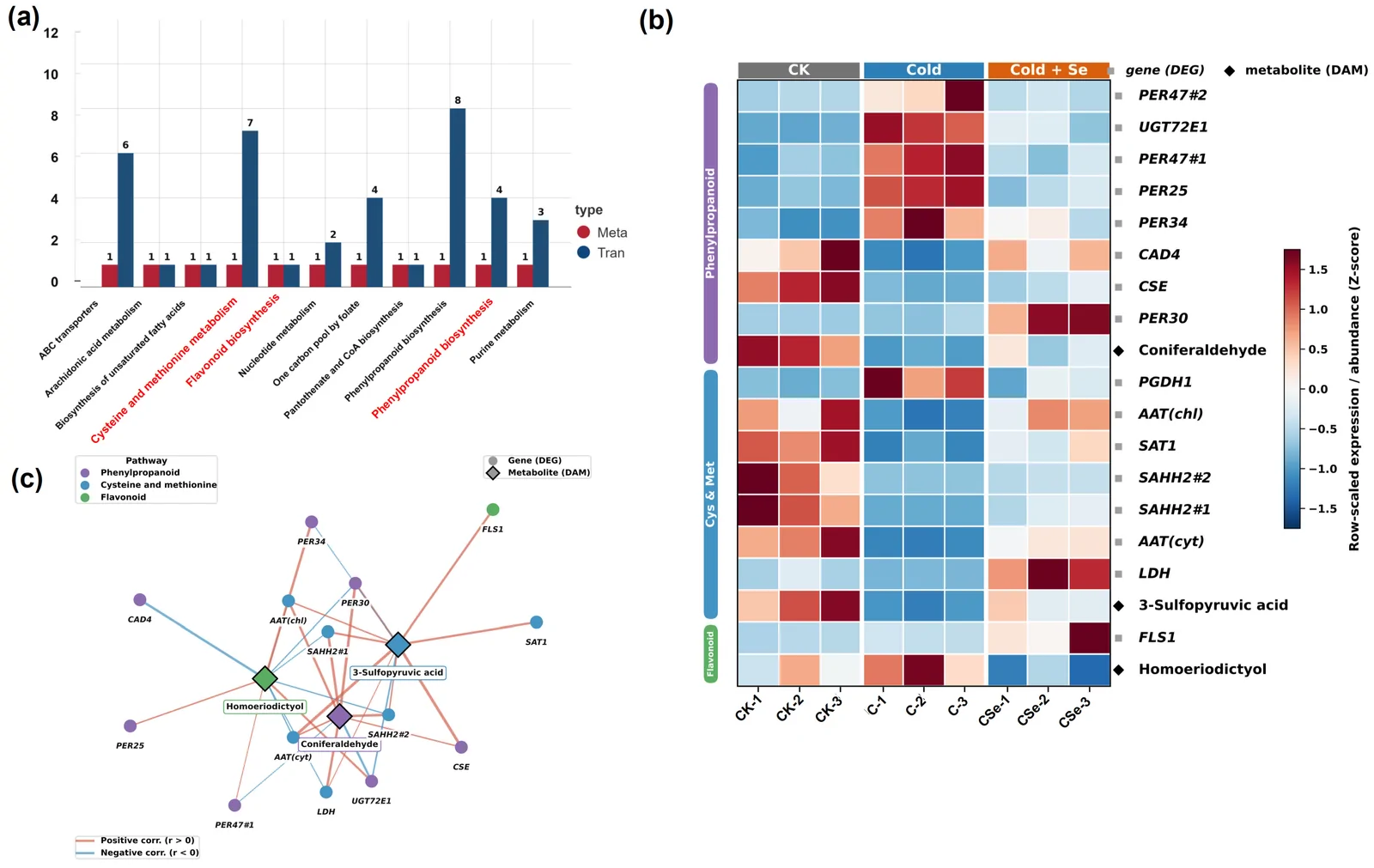
Integrated transcriptomic and metabolomic analysis of molecular changes associated with sodium selenite treatment under cold stress (CSe vs. C). (**a**) Numbers of DEGs (Tran) and DAMs (Meta) co-enriched in the KEGG pathways shared between the transcriptome and metabolome. The three pathways selected for focused exploratory cross-omics visualization—cysteine and methionine metabolism, flavonoid biosynthesis, and phenylpropanoid biosynthesis—are highlighted in red. (**b**) Heatmap of the selected genes and metabolite features assigned to the three pathways across CK, C, and CSe. The features were selected from the CSe versus C comparison and were then displayed across all nine samples. Samples are shown in a fixed order as CK1 to CK3, C1 to C3, and CSe1 to CSe3. Squares and diamonds denote genes and metabolite features, respectively. The colored bars on the left indicate pathway assignment. (**c**) Correlation network between the DEGs and DAMs of the three pathways. Pairwise Pearson correlation coefficients were calculated across the six samples (three C and three CSe replicates), and only pairs with |*r*| ≥ 0.8 and *p* < 0.05 are shown as edges. Circles are genes (Gene, DEG) and diamonds are metabolite features (Metabolite, DAM); node color denotes the pathway (purple, phenylpropanoid; blue, cysteine and methionine; green, flavonoid). Red edges indicate a positive correlation (*r* > 0) and blue edges a negative correlation (*r* < 0); the edge width is proportional to |*r*|. *CAD4*, cinnamyl alcohol dehydrogenase 4; *CSE*, caffeoylshikimate esterase; *PER*, peroxidase; *UGT72E1*, UDP-glycosyltransferase 72E1; *AAT* (chl) *and AAT* (cyt), genes encoding chloroplastic and cytosolic aspartate aminotransferases, respectively; *SAT1*, serine acetyltransferase 1; *SAHH2*, S-adenosylhomocysteine hydrolase 2; *PGDH1*, phosphoglycerate dehydrogenase 1; *LDH*, lactate dehydrogenase; *FLS1*, flavonol synthase 1. Coniferaldehyde was putatively annotated at Level 2, the feature assigned to 3-sulfopyruvic acid was tentatively annotated at Level 3, and homoeriodictyol was confirmed using an authentic reference standard at Level 1. C, 4 °C cold stress; CSe, cold stress supplemented with 20 mg L^−1^ Na_2_SeO_3_.

To identify pathways represented in both omics datasets, DEGs and candidate DAMs from the CSe vs. C comparison were mapped to the KEGG database. Pathways represented in both datasets were considered for focused exploratory analysis. Cysteine and methionine metabolism and phenylpropanoid biosynthesis were prioritized because each contained multiple DEGs together with a candidate DAM and directly addressed the biological focus of the study. Flavonoid biosynthesis was retained as a secondary, biochemically related branch, although it was represented by only one DEG and one candidate DAM (Figure 5a). This selection was used to organize the integrated results and was not treated as an independent statistical ranking of pathway importance.

The complete list of selected features and their sample-level values is provided in Table S3. In phenylpropanoid biosynthesis, *PER47#1*, *PER47#2*, *PER25*, *PER34*, and *UGT72E1* showed higher relative expression in C than in CK and showed lower expression in CSe than in C. *CAD4* and *CSE* shifted in the opposite direction and expression in CSe was closer to that in CK, while *PER30* reached its highest expression in CSe. The putatively annotated coniferaldehyde feature also showed a partial shift toward the CK pattern. In cysteine and methionine metabolism, several *AAT*, *SAT1*, and *SAHH2* transcripts showed higher expression in CSe than in C and expression in CSe was closer to that in CK. The feature tentatively assigned to 3-sulfopyruvic acid showed a similar trend. *LDH* reached its highest expression in CSe, whereas *PGDH1* was higher in C. The flavonoid branch was less consistent: *FLS1* was highest in CSe, whereas homoeriodictyol declined to below the CK level (Figure 5b).

An exploratory correlation network was used to summarize pairwise associations between the selected metabolite features and genes that differed between CSe and C. All retained edges and their corresponding Pearson coefficients and *p*-values are provided in Table S4. Among the three selected metabolite features, the feature tentatively assigned to 3-sulfopyruvic acid had the largest number of retained gene correlations. It was positively correlated with *AAT*, *SAT1*, *SAHH2*, *CSE*, and *PER30*. The feature putatively annotated as coniferaldehyde showed a broadly similar correlation pattern. In contrast, homoeriodictyol was positively correlated with several peroxidase genes and *UGT72E1*, but negatively correlated with several sulfur-related genes (Figure 5c).

## 3. Discussion

Selenium (Se) is a beneficial element whose effects on plant growth and stress responses are strongly concentration-dependent [13,20]. In this study, cold stress (4 °C) reduced whole-seedling fresh weight (Figure 1b) and caused a visible reduction in seedling size (Figure 1a). Foliar Na_2_SeO_3_ partially restored fresh weight, but the response was not proportional to the applied concentration. Among the concentrations tested, C+20 showed the greatest increase in fresh weight relative to C. Responses depending on Se dose or formulation have also been reported in *Brassica napus* and *Brassica rapa* under other abiotic stresses [21,22]. In wheat seedlings, low Se doses increased biomass under cold stress, whereas higher doses did not further promote biomass accumulation [23]. Se supply also improved seedling growth under cold stress in buckwheat [24]. These observations are consistent with the dose-dependent action of Se, in which an appropriate concentration may alleviate stress-associated growth inhibition, whereas a higher concentration may provide no additional benefit [20]. These comparisons support the view that 20 mg L^−1^ was the most effective concentration within the tested range, rather than a universal optimum for rapeseed. Fresh weight was used as a rapid whole-seedling endpoint and is influenced by both tissue hydration and dry-matter accumulation. The partial restoration of fresh weight therefore indicates an improvement in fresh weight, but it does not by itself establish increased dry biomass or improved water status.

The concentration-dependent growth response was accompanied by corresponding changes in oxidative status. In this study, cold stress increased O_2_^−^ production, H_2_O_2_ and MDA contents, and the activities of SOD, POD, and CAT (Figure 2). This pattern suggests that cold imposed a strong oxidative challenge and activated enzymatic antioxidant responses. However, the high ROS and MDA levels indicate that these responses did not fully prevent oxidative damage. A similar increase in H_2_O_2_ and MDA have been reported in citrus seedlings under cold stress [25]. Relative to C treatment, C+20 showed the lowest ROS and MDA levels and lower activities of all three antioxidant enzymes. The concurrently lower ROS and MDA levels and antioxidant enzyme activities are compatible with a lower oxidative challenge at the sampling endpoint. They do not establish whether the lower ROS levels reduced the demand for antioxidant enzyme activity or resulted from enzymatic scavenging. This distinction is important because Se can increase antioxidant-enzyme activity in other stress systems [26], whereas Se treatment under low temperature reduced H_2_O_2_ and MDA but produced a different enzyme response in tea [27]. Such variation indicates that antioxidant responses depend on plant species, Se dose, stress severity, and sampling time [28,29]. The partial rebound of O_2_^−^, H_2_O_2_, and MDA in C+40 further shows that the higher tested concentration of 40 mg L^−1^ did not further improve the oxidative-stress indicators under the present conditions. Taken together, the fresh-weight and oxidative-status results supported the selection of C+20 for subsequent transcriptomic and metabolomic analyses.

The enrichment of photosynthesis- and carbohydrate-related processes in the C vs. CK and CSe vs. CK comparisons (Figures S1 and S2) may reflect the overall response to 4 °C under the maintained light regime, although a contribution from excess excitation under cold conditions cannot be excluded. Because low temperature limits the photosynthetic use of absorbed light energy, the same incident PPFD may impose greater excitation pressure than under normal-temperature conditions [30,31,32]. Nevertheless, the C and CSe groups were exposed to identical temperature and light conditions, and their comparison therefore remains informative for identifying Se-associated responses under cold conditions.

At the molecular level, Se treatment did not simply restore cold-stressed seedlings to the CK state. Cold stress altered the expression of 15,397 genes, whereas only about 5% of the cold-responsive genes returned to the CK range or moved toward CK in CSe (Figure S6a). Most cold-responsive genes showed no statistically detectable difference between C and CSe under the applied criteria, and 456 genes differed between CSe and C without being identified as cold-responsive. The metabolome showed the same overall pattern. Only 3 of the 76 cold-responsive metabolites returned to the CK range, whereas 71 metabolites differed between CSe and C but not between C and CK (Figure S6b). Under the applied screening criteria, the limited overlap among the candidate DAM sets in Figure 4b reflects the same comparison-specific pattern summarized in Figure S6b. Moreover, the separation of CSe from both CK and C in the PCA (Figure 4a) indicates that the CSe metabolome remained distinct rather than broadly converging toward the CK profile. These molecular state coincided with lower ROS and MDA levels and improved fresh weight, but the concurrent design does not establish that the molecular changes caused the physiological response [33,34].

The transcriptomic enrichment results provide a broader functional context for this selective response. In the CSe vs. C comparison, DEGs were enriched in GO terms related to the cell wall and apoplast, glucan and polysaccharide metabolic processes, and glucosyltransferase and cellulose synthase activities. Enriched KEGG pathways included starch and sucrose metabolism, pentose and glucuronate interconversions, ABC transporters, and arginine and proline metabolism (Figure 3c,d). Starch and sucrose metabolism and pentose and glucuronate interconversions can supply carbohydrates for cell-wall metabolism [35]. Arginine and proline metabolism is linked to osmotic adjustment at low temperature [36], while ABC transporters may contribute to solute transport. These terms point to coordinated changes in carbon allocation, solute transport, osmotic adjustment, and cell-wall-related metabolism. However, these enrichment results do not demonstrate cell-wall reinforcement because cell-wall composition and structure were not measured. Whether the observed transcriptional changes translate into cell-wall remodeling requires further investigation. This pathway-level background guided the integrated analysis toward processes supported by both the transcriptomic and metabolomic datasets.

Among the pathways represented in both omics datasets, phenylpropanoid biosynthesis and cysteine and methionine metabolism contained the largest sets of selected features, whereas flavonoid biosynthesis was represented by only one gene and one metabolite (Figure 5a). Among the selected pathways, cysteine and methionine metabolism showed the most internally consistent cross-omics pattern associated with sodium selenite treatment. *AAT* (chl), *AAT* (cyt), *SAT1*, *SAHH2#1*, and *SAHH2#2* showed lower relative expression in C than in CK. Their expression levels were higher in CSe than in C and closer to those in CK. The feature tentatively assigned to 3-sulfopyruvic acid showed a similar trend. *LDH* reached its highest expression in CSe, whereas *PGDH1* expression was higher in C than in CSe (Figure 5b). These patterns are biologically plausible because SAT contributes to O-acetylserine formation, SAHH supports methyl-cycle turnover, and Se metabolism intersects with sulfur-containing amino acid pathways [15,16,37]. Recent work in *Cardamine hupingshanensis* identified responses involving *SAT* and *OASTL* in Se metabolism [38], and integrated analyses in tobacco also linked Se treatment with sulfur-related metabolic changes [39]. Together, these data show coordinated treatment-associated changes in sulfur-related and methyl-cycle transcripts and in the feature tentatively assigned to 3-sulfopyruvic acid. Selenite and selenate differ in their initial uptake and conversion routes [15]. Because Se uptake, tissue distribution, and chemical speciation were not measured, and key pathway intermediates and metabolic flux were not directly quantified, these results support pathway-level associations rather than confirmed metabolic restructuring. The changes in *PGDH1* and the nominal enrichment of one carbon pool by folate suggest a possible connection with serine and one-carbon metabolism [37,40,41], but this branch was supported by limited metabolite evidence and requires further validation.

Phenylpropanoid-related features showed heterogeneous treatment-associated patterns rather than uniform pathway activation. *PER47#1*, *PER47#2*, *PER25*, *PER34*, and *UGT72E1* showed higher expression in C than in CK and lower expression in CSe than in C, whereas *CAD4*, *CSE*, and the feature putatively annotated as coniferaldehyde showed the opposite pattern. *PER30* differed from both groups and reached its highest expression in CSe (Figure 5b). CAD and CSE participate in monolignol biosynthesis [42,43]. Previous studies have associated Se treatment and cold tolerance with processes related to lignin and the cell wall [44,45,46]. In the present study, however, the treatment was associated with changes in *CAD4*, *CSE*, several *PER* transcripts, and the putatively annotated coniferaldehyde feature. These molecular features are related to phenylpropanoid metabolism and monolignol biosynthesis. They do not demonstrate increased lignin synthesis, lignin deposition, or cell-wall reinforcement because lignin content and cell-wall composition were not measured. Lower expression of several class III peroxidase genes in CSe than in C coincided with lower POD activity and H_2_O_2_ content. This agreement across transcript and physiological measurements indicates coordinated responses at the sampling endpoint [47], not a defined direction of regulation. The flavonoid branch was less consistent: *FLS1* was highest in CSe, whereas homoeriodictyol abundance was higher in C than in CK but lower in CSe than in both C and CK. Because this pathway was represented by only one selected gene and one metabolite with opposing patterns, it should remain a secondary interpretation. The exploratory correlation network summarized coordinated variation among the selected sulfur-related and phenylpropanoid features. However, it was constructed from only six samples in two treatment groups, so strong correlations may partly reflect treatment separation rather than direct gene-metabolite regulation.

**Figure 6:**
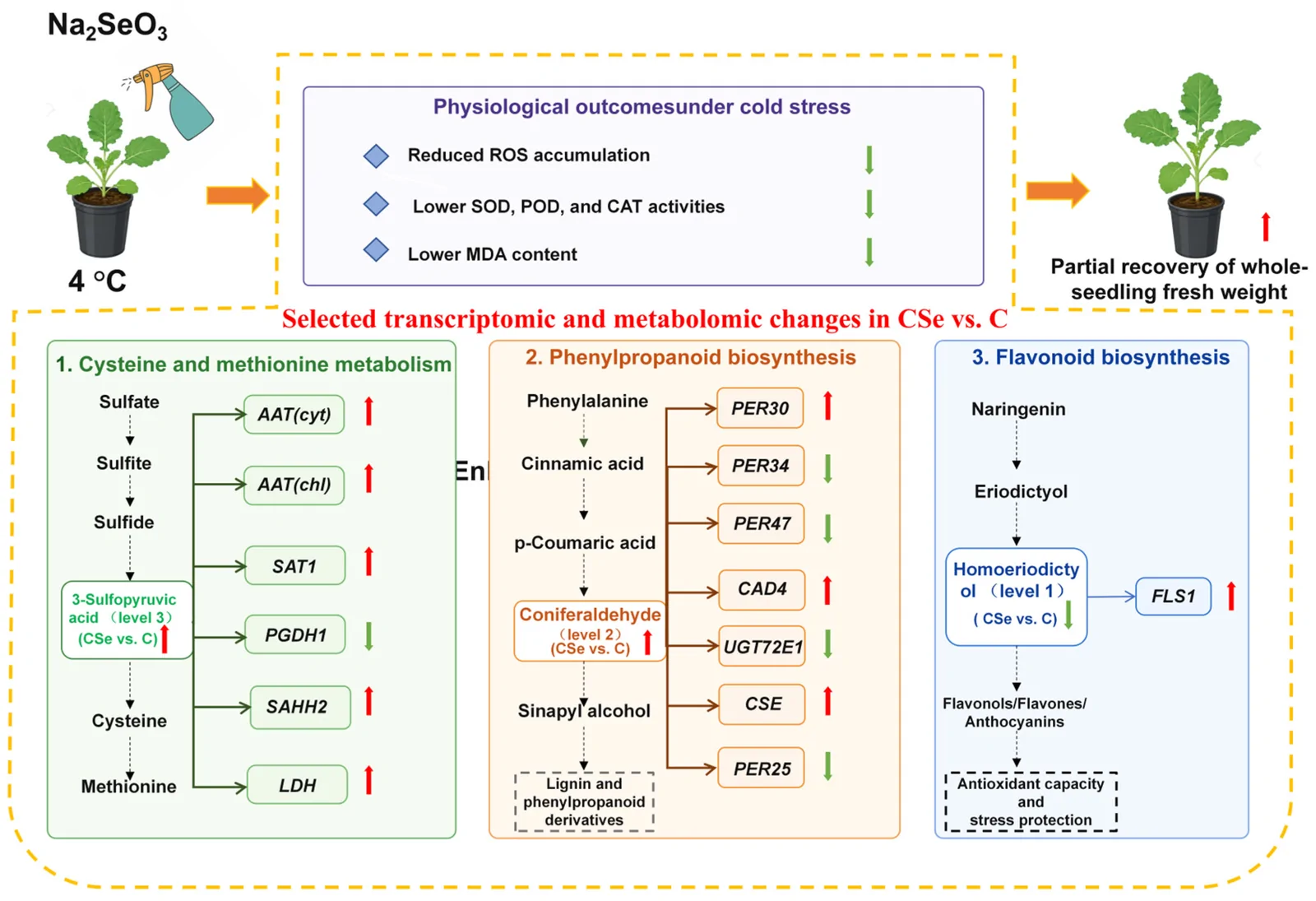
Association-based working model of physiological and molecular changes associated with foliar sodium selenite treatment under cold stress in rapeseed. The model summarizes experimentally measured differences between the CSe treatment and the cold-stressed control (C). CSe represents seedlings exposed to 4 °C and supplemented with 20 mg L^−1^ Na_2_SeO_3_. Relative to C, CSe showed higher whole-seedling fresh weight, with the mean value approaching that of CK at the 3-d endpoint, together with lower superoxide anion production, H_2_O_2_ and MDA contents, and SOD, POD, and CAT activities. The concurrent decreases in ROS, MDA, and antioxidant-enzyme activities are compatible with a lower oxidative challenge at the sampling endpoint; they do not establish the temporal order or causal relationship among these variables. The lower part of the model summarizes selected transcriptomic and metabolomic features assigned to cysteine and methionine metabolism, phenylpropanoid biosynthesis, and flavonoid biosynthesis. Red upward and green downward arrows indicate higher and lower values, respectively, in CSe relative to C. The feature assigned to 3-sulfopyruvic acid was tentatively annotated at Level 3, coniferaldehyde was putatively annotated at Level 2, and homoeriodictyol was identified at Level 1 using an authentic reference standard. Dashed arrows provide simplified biochemical or pathway context and do not indicate experimentally verified metabolic flux. Potential downstream outcomes were not measured in this study. Connections between gene boxes and pathway components indicate pathway assignment rather than direct regulation. The model summarizes concurrent, treatment-associated changes and should not be interpreted as a confirmed causal pathway, evidence of enhanced lignification, or proof of a cold-specific Se response. *AAT* (chl) *and AAT* (cyt), genes encoding chloroplastic and cytosolic aspartate aminotransferases, respectively; *SAT1*, serine acetyltransferase 1; *PGDH1*, phosphoglycerate dehydrogenase 1; *SAHH2*, S-adenosylhomocysteine hydrolase 2; *LDH*, lactate dehydrogenase; *CAD4*, cinnamyl alcohol dehydrogenase 4; *CSE*, caffeoylshikimate esterase; *PER*, peroxidase; *UGT72E1*, UDP-glycosyltransferase 72E1; *FLS1*, flavonol synthase 1; ROS, reactive oxygen species; MDA, malondialdehyde; SOD, superoxide dismutase; POD, peroxidase; CAT, catalase; C, cold stress at 4 °C; CSe, cold stress at 4 °C supplemented with 20 mg L^−1^ Na_2_SeO_3_.

Taken together, the data show that 20 mg L^−1^ Na_2_SeO_3_ was associated with the most favorable physiological response among the cold-treated groups within the tested range, while the molecular response was selective rather than restorative. The most coherent cross-omics changes involved sulfur-related and methyl-cycle features together with phenylpropanoid- and peroxidase-related processes. Figure 6 therefore summarizes an association-based working model rather than a confirmed causal sequence. Only samples from C+20 were subjected to transcriptomic and metabolomic analyses, so the molecular basis of the physiological dose response and the weaker performance of C+40 cannot be resolved. The absence of a normal-temperature Se treatment prevents separation of general Se responses from responses that depend on cold conditions. All physiological and omics variables were measured at a single endpoint, and the candidate metabolite and pathway analyses were exploratory. In addition, the selected metabolites and metabolite features had different identification confidence levels. Accordingly, the present results define a testable set of physiological and molecular associations but do not resolve temporal order, metabolic flux, or direct causality. Future studies should include omics analyses across multiple Se concentrations, a Se-treated normal-temperature control, time-course sampling, and independent validation. Additional priorities include assessing Se uptake and speciation, quantifying metabolites involved in sulfur metabolism and the methyl cycle, measuring lignin and cell-wall traits, and functionally validating key genes such as *SAT1*, *SAHH2*, *CAD4*, and *CSE*.

## 4. Materials and Methods

### 4.1. Plant Material and Treatments

Seeds of rapeseed (*Brassica napus* L. cv. Zhongshuang No. 11) were surface-sterilized in 75% (*v*/*v*) ethanol for 30 s and rinsed five times with sterile distilled water. The seeds were germinated on moist gauze in a controlled-environment incubator under a 16 h light/8 h dark photoperiod. The temperature was maintained at 25 °C during the light period and 18 °C during the dark period, and the photosynthetic photon flux density (PPFD) was 392–415 μmol photons m^−2^ s^−1^. After 10 d, uniform seedlings were transferred to plastic boxes containing half-strength Hoagland nutrient solution prepared with purified water and were grown under the same light and temperature regime until the six- to seven-leaf stage. The seedlings were maintained hydroponically without separate irrigation, and the nutrient solution was renewed daily throughout the growth and treatment periods.

Five treatments were established: CK, maintained under the normal-temperature regime without Se; C, exposed to 4 °C without Se; and C+10, C+20, and C+40, exposed to 4 °C and foliar-sprayed with 10, 20, and 40 mg L^−1^ Na_2_SeO_3_, respectively. The foliar treatments were applied once, immediately before the plants were exposed to cold stress. The concentration range was selected with reference to recent studies evaluating Na_2_SeO_3_ application in *Brassica* species and foliar Se application in other crops [48,49,50]. The experiment followed a completely randomized design with three box-level biological replicates per treatment. Each replicate consisted of a separate box containing 12 seedlings, giving a total of 36 seedlings per treatment. The box was used as the unit of biological replication and statistical analysis. Seedlings within the same box were treated as subsamples and were not counted as independent biological replicates. Approximately 5 mL of spray solution was applied per plant, corresponding to approximately 60 mL per box of 12 plants, until all leaf surfaces were uniformly wetted. The CK and C plants received a mock spray of purified water, whereas the C+10, C+20, and C+40 plants received the corresponding Na_2_SeO_3_ solutions. All spray solutions, including the mock controls, contained 0.01% (*v*/*v*) Tween-20. The same spray volume and application procedure were used for all treatment groups. After spraying, the CK plants remained under the normal temperature regime of 25 °C during the light period and 18 °C during the dark period. The C, C+10, C+20, and C+40 boxes were transferred directly to a chamber maintained at 4 °C for 3 d. During the cold treatment, the photoperiod and PPFD were maintained at the same settings used during the preceding growth period, namely 16 h light/8 h dark and 392–415 μmol photons m^−2^ s^−1^. The cold-treated plants were maintained at 4 °C throughout both the light and dark periods. Thus, CK and C received the same incident-light regime but differed in temperature, whereas all cold-treated groups shared the same temperature and light conditions. The 3-d treatment was used as a short-term endpoint for physiological and multi-omics profiling during continuous cold exposure.

At the end of the 3-d treatment, the seedlings were rinsed first with tap water and then with distilled water, gently blotted dry, and immediately weighed. Whole-seedling fresh weight was recorded, after which the samples were immediately frozen in liquid nitrogen and stored at −80 °C. Whole-seedling material collected at this endpoint was used for all physiological, transcriptomic, and metabolomic analyses. This provided a common tissue basis across datasets and allowed characterization of the integrated seedling response, but did not resolve organ-specific effects.

### 4.2. Measurement of Antioxidant Enzyme Activities

Approximately 0.10 g of fresh whole-seedling tissue was homogenized in 1.0 mL of pre-cooled 50 mM phosphate buffer (pH 7.0). The homogenate was centrifuged at 12,000× *g* for 15 min at 4 °C, and the supernatant was used for the assays. Superoxide dismutase (SOD) activity was measured by the nitroblue tetrazolium (NBT) method [51]. The absorbance of the reaction mixture was recorded at 560 nm, and one unit (U) of enzyme activity was defined as the amount causing 50% inhibition of NBT photoreduction. Peroxidase (POD) activity was measured by the guaiacol method at 470 nm [52]. One unit (U) of POD activity was defined as the amount of enzyme causing an increase of 0.005 in absorbance at 470 nm per minute under the assay conditions. Catalase (CAT) activity was assayed by mixing 100 µL of supernatant with 2.9 mL of 20 mM H_2_O_2_ and recording the absorbance change at 240 nm over 1 min at 25 °C, with one unit defined as a change of 0.1 absorbance unit per minute [53]. All values were expressed on a fresh-weight basis.

### 4.3. Measurement of Malondialdehyde Content and Reactive Oxygen Species

Malondialdehyde (MDA) content was determined by the thiobarbituric acid (TBA) method [54]. About 0.1 g of fresh whole-seedling tissue was homogenized in 5% (*w*/*v*) trichloroacetic acid (TCA) and centrifuged at 12,000× *g* for 10 min. An aliquot of the supernatant was mixed with an equal volume of 0.5% (*w*/*v*) TBA in 20% TCA, heated at 95 °C for 30 min, cooled on ice and centrifuged again. Absorbance was measured at 450, 532, and 600 nm using a spectrophotometer. The superoxide anion (O_2_^−^) production rate was determined as described previously [55]. The hydrogen peroxide (H_2_O_2_) content was determined as described previously [56].

Based on the physiological measurements, C+20 showed the highest whole-seedling fresh weight and the lowest O_2_^−^ production rate and H_2_O_2_ and MDA contents among the cold-treated groups at the 3-d endpoint. The C+20 group was therefore selected for omics analyses and is hereafter designated CSe; whole-seedling samples from CK, C, and CSe were subjected to transcriptomic and metabolomic analyses.

### 4.4. RNA Extraction and Transcriptome Sequencing

Total RNA was extracted from whole-seedling samples of the three groups (nine samples, three replicates per group) using the RNAprep Pure Plant Kit (TIANGEN, Beijing, China). RNA purity and concentration were checked with a NanoDrop ND-2000 spectrophotometer (Thermo Fisher Scientific, Wilmington, DE, USA), and RNA integrity was checked with an Agilent 2100 Bioanalyzer (Agilent Technologies, Santa Clara, CA, USA). Each RNA extract submitted for library preparation had a volume of 45 μL, and RNA concentrations ranged from 14–402 ng μL^−1^. For each library, 200 ng of total RNA was used as input, and only samples meeting the library-preparation quality criteria were processed. Poly(A)+ mRNA was enriched using oligo(dT)-coupled magnetic beads, thereby excluding most rRNA and other non-polyadenylated RNA species. The enriched mRNA was used for library construction. The constructed libraries were quantified using a Qubit 2.0 Fluorometer (Life Technologies, Carlsbad, CA, USA), and their insert sizes were assessed using an Agilent 2100 Bioanalyzer. The effective library concentrations were further determined by quantitative PCR, and qualified libraries were pooled and sequenced on an Illumina NovaSeq 6000 platform (Illumina Inc., San Diego, CA, USA) to generate 150-bp paired-end reads.

Raw reads were filtered using fastp (v0.23) to remove adapter sequences and low-quality reads. The resulting clean paired-end reads were aligned to the *Brassica napus* cv. Zhongshuang No. 11 (ZS11) reference genome (BnIR: ZS11.v10) using HISAT2 (v2.0.5) with default parameters. Gene-level read counts were generated using featureCounts (v1.5.0-p3) based on the corresponding ZS11 genome annotation. Reads with mapping quality scores below 10, unpaired alignments, and reads mapped to multiple genomic locations were excluded. All R-based transcriptomic analyses were performed in R (v3.5.0). The resulting raw count matrix was used for downstream differential expression analysis. The raw data were deposited in the NCBI Sequence Read Archive under accession number PRJNA1481826.

### 4.5. Identification and Enrichment Analysis of Differentially Expressed Genes (DEGs)

DEGs were identified with DESeq2 (v1.20.0), using |log_2_(fold change)| ≥ 1 and an adjusted *p*-value < 0.05 as the thresholds [19]. Three pairwise comparisons were performed: C vs. CK, CSe vs. CK and CSe vs. C. A Venn diagram showing shared and unique DEGs among the three comparisons was generated using the VennDiagram package (v1.6.20) in R (v3.5.0). Gene Ontology (GO) and Kyoto Encyclopedia of Genes and Genomes (KEGG) over-representation analyses were performed using clusterProfiler (v3.8.1) [57]. In the GO enrichment, the DEGs were divided into molecular function (MF), biological process (BP) and cellular component (CC) categories. The resulting *p*-values were adjusted for multiple testing using the Benjamini–Hochberg (BH) procedure. GO terms and KEGG pathways with a BH-adjusted *p*-value < 0.05 were considered significantly enriched.

### 4.6. qRT-PCR Validation

The same whole-seedling RNA samples used for RNA-seq were used for qRT-PCR validation. Nine DEGs representing key pathways were selected to verify the expression trends observed by RNA-seq. Primer sequences for the selected DEGs are provided in Table S5. Total RNA was reverse-transcribed into cDNA with the HiScript III 1st Strand cDNA Synthesis Kit (Vazyme, Nanjing, China). qRT-PCR was performed using a real-time PCR system with a SYBR Green master mix, using the *Brassica napus ACTIN* gene as the internal reference. The thermal cycling program consisted of 95 °C for 30 s, followed by 40 cycles of 95 °C for 10 s and 60 °C for 30 s. Three technical replicates were run for each of the three biological replicates. Relative expression levels were calculated using the 2^−ΔΔCT^ method.

### 4.7. Metabolite Extraction and Metabolome Analysis

The same three groups (CK, C and CSe; three replicates per group) were used for metabolite profiling. Approximately 100 mg of frozen, finely ground whole-seedling tissue from each biological replicate was extracted with 500 μL of 80% (*v*/*v*) methanol. The samples were vortexed, incubated in an ice bath for 5 min, and centrifuged at 15,000× *g* for 20 min at 4 °C. A procedural blank consisting of 53% (*v*/*v*) aqueous methanol and containing no plant material was processed in parallel through the same vortexing, ice-bath incubation, and centrifugation steps and was used to identify and exclude background signals. The pooled QC samples were used to equilibrate the LC–MS system and evaluate analytical stability throughout the analytical sequence. Metabolites were analyzed on a Vanquish UHPLC system coupled to a Q Exactive HF mass spectrometer (Thermo Fisher Scientific, Munich, Germany). Chromatographic separation was performed on a Hypersil Gold C18 column (100 × 2.1 mm, 1.9 µm; Thermo Fisher Scientific, Wilmington, DE, USA). The column temperature was maintained at 40 °C, and the flow rate was 0.2 mL min^−1^. Mobile phase A consisted of LC–MS-grade water containing 0.1% (*v*/*v*) formic acid, and mobile phase B consisted of LC–MS-grade methanol. The gradient program was as follows: 0–1.5 min, 2% B; 3 min, 85% B; 10 min, 100% B; and 10.1–12 min, 2% B. Mass spectra were acquired using an electrospray ionization source in both positive and negative ion modes over an m/z range of 100–1500. The spray voltage was set to 3.5 kV. The sheath-gas and auxiliary-gas settings were 35 and 10, respectively. The capillary temperature was maintained at 320 °C, the auxiliary-gas heater temperature was maintained at 350 °C, and the S-lens RF level was set to 60. The declustering voltage was set to 3.2 V. Full MS scans were acquired at a resolution of 60,000 FWHM, with an automatic gain control target of 3 × 10^6^ and a maximum injection time of 100 ms. Data-dependent MS/MS spectra were acquired at a resolution of 15,000 FWHM, with an automatic gain control target of 2 × 10^5^ and a maximum injection time of 25 ms. Stepped normalized collision energies of 20, 40, and 60 were applied within each MS/MS acquisition event.

Raw data were processed using Compound Discoverer (3.3). The processing workflow included retention-time alignment, peak detection, peak-area integration, and feature quantification. Peak areas were corrected using the first QC injection, and signals detected in the blank samples were removed. Relative peak areas were normalized according to the following equation: raw peak area/(total peak area of the sample/total peak area of QC1). Peak extraction was performed using a mass tolerance of 5 ppm and an intensity tolerance of 30%. Molecular formulas were predicted from precursor and product ions. Metabolite features were annotated by matching the experimental mass spectra against the mzCloud, mzVault, and MassList databases. Features with a coefficient of variation above 30% across the QC injections were excluded from subsequent analysis. Representative total ion chromatograms of the pooled QC samples acquired in positive and negative ion modes are provided in Figure S7. Metabolite identification confidence was assigned according to the available analytical evidence. Level 1 indicated identification confirmed using an authentic reference standard based on agreement in retention time, precursor mass, and MS/MS fragmentation. Level 2 indicated a putative compound annotation supported by accurate mass and an MS/MS library match but without an authentic reference standard. Level 3 indicated a tentative assignment based on accurate mass, molecular formula, and database information when neither an authentic standard nor a matched reference-library MS/MS spectrum was available. The metabolomic raw data were deposited in the OMIX database of the China National Center for Bioinformation under accession number OMIX018860.

### 4.8. Identification and Enrichment Analysis of Differentially Accumulated Metabolites (DAMs)

The resulting feature table was then imported into metaX for downstream statistical analysis [58]. Principal component analysis (PCA) was used to compare the CK, C and CSe groups. Pairwise *p* values were calculated using Student’s *t*-test, and fold change (FC) was calculated from the mean normalized abundance of the two groups. For each comparison, FC was expressed as the first-named group relative to the second-named group. Thus, for the CSe vs. C comparison, FC was calculated as the mean normalized abundance in CSe divided by that in C, and log_2_FC was calculated as log_2_(CSe/C).

Metabolite features meeting the predefined exploratory criteria of VIP > 1, nominal *p* < 0.05, and FC ≥ 1.5 or FC ≤ 0.67 were retained as candidate DAMs for subsequent exploratory analyses. All R-based metabolomic visualizations were performed in R (v3.5.0). The shared and unique candidate DAMs among the three comparisons were visualized using the VennDiagram R package. Principal component analysis and volcano plots were generated using ggplot2. The relative abundance patterns of the candidate DAMs were visualized in a heatmap generated using the pheatmap R package after row-wise z-score normalization. Hierarchical clustering was performed using Pearson correlation-based distance and complete linkage. The candidate DAMs were mapped to the KEGG database and subjected to pathway over-representation analysis. Pathways with nominal *p* values below 0.05 were displayed in bubble plots.

### 4.9. Classification of Molecular Response Patterns Across CK, C, and CSe

To distinguish cold-responsive features from changes associated with Se treatment under cold conditions, genes and candidate metabolite features were first evaluated in the C versus CK comparison. Fold change directions were expressed as C relative to CK, Cse relative to C, and Cse relative to CK. The same statistical thresholds used in the original DEG and DAM analyses were applied to all three comparisons.

Cold-responsive features were assigned to four response classes. A feature was classified as returned to the CK range when it was significant in both C vs. CK and Cse vs. C, and the two changes were in opposite directions. The feature was also required to show no significant difference between Cse and CK. A feature was classified as moved toward CK but remained different when the changes in C versus CK and Cse versus C were significant and opposite in direction. The feature remained significantly different between Cse and CK. Its direction relative to CK was the same as that observed under cold stress, but the absolute log_2_ fold change was smaller than that in C versus CK. A feature was classified as changed further in the same direction when the changes in C versus CK and Cse versus C were both significant and had the same direction. The absolute log_2_ fold change relative to CK was also greater in Cse than in C. A feature was classified as showing no detectable response to Se when it differed significantly between C and CK but did not differ significantly between Cse and C.

Features that differed significantly between Cse and C but not between C and CK were reported separately as changes associated with Se treatment under cold conditions. These features were not considered evidence of reversal of the cold response. Percentages in Figure S6 were calculated using the total number of cold-responsive features identified in C versus CK as the denominator. The separately reported features were not included in this denominator.

The term returned to the CK range indicates that a feature no longer met the differential threshold in the Cse versus CK comparison. It does not indicate formal statistical equivalence between Cse and CK.

### 4.10. Integrated Transcriptomic and Metabolomic Analysis

DEGs and candidate DAMs from the Cse vs. C comparison were mapped to the KEGG database. Pathways represented in both datasets were identified. Phenylpropanoid biosynthesis, cysteine and methionine metabolism, and flavonoid biosynthesis were selected for further examination because each pathway contained both transcriptomic and metabolomic features. To place the Cse vs. C differences in the context of the cold response, the selected features were displayed across CK, C, and Cse. Normalized gene expression values and normalized metabolite peak areas were extracted for all nine samples. Each feature was standardized across the nine samples by row-wise Z-score transformation. Samples were shown in a fixed order as CK1-CK3, C1-C3, and Cse1-Cse3. Features were grouped according to their KEGG pathways. The heatmap was used to compare the relative pattern of each feature across treatments. It was not used for statistical significance testing. The selected features, their annotations, and sample-level values are provided in Table S3.

Pairwise Pearson correlation coefficients were calculated between the selected genes and metabolites using the three C samples and three Cse samples. The CK samples were included in the heatmap but were not included in the correlation analysis because the network was designed to explore covariation within the Cse vs. C comparison. Gene-metabolite pairs with an absolute Pearson correlation coefficient of at least 0.8 and a nominal *p*-value below 0.05 were retained as network edges. All retained gene–metabolite pairs and their corresponding Pearson correlation coefficients (*r*) and nominal *p* values are provided in Table S4. Genes and metabolites were shown as circles and diamonds, respectively. Node colors indicated pathway assignment. Red and blue edges indicated positive and negative correlations. Edge width was proportional to the absolute correlation coefficient. The network was visualized using Gephi (v0.9.2).

### 4.11. Statistical Analysis

Data are expressed as the mean ± standard deviation (SD) of three independent biological replicates (*n* = 3). The normality assumption for each variable was assessed using the Shapiro–Wilk test, and homogeneity of variance among treatments was evaluated using Levene’s test. Data were subjected to one-way ANOVA followed by Duncan’s multiple range test, with *p* < 0.05 considered statistically significant. All statistical analyses were performed using SPSS Statistics v26.0 (IBM, Armonk, NY, USA), and physiological bar plots were prepared using OriginPro 2026 (OriginLab, Northampton, MA, USA). Different lowercase letters above the bars indicate the results of the post hoc multiple-comparison test. Means sharing at least one letter are not significantly different, whereas means with no letter in common differ significantly.

## 5. Conclusions

In summary, after 3 d of continuous cold exposure, 20 mg L^−1^ Na_2_SeO_3_ was associated with the highest whole-seedling fresh weight and the lowest ROS and MDA levels among the cold treatments. These physiological differences coincided with selective changes in transcripts and candidate metabolite features assigned to sulfur metabolism, the methyl cycle, and phenylpropanoid biosynthesis, together with changes in several peroxidase transcripts. Terms related to photosynthesis and carbohydrate metabolism were also enriched in both C vs. CK and CSe vs. CK. Because these enrichments were not specific to the CSe vs. C comparison and photosynthetic performance was not measured directly, they should be interpreted as part of the overall response to 4 °C under the maintained light regime rather than as evidence that Se directly improved photosynthesis. The single-endpoint design does not resolve temporal order, causality, metabolic flux, lignin accumulation, or cell-wall reinforcement. The results therefore support a working model based on associations and identify priorities for future studies, including time course sampling, direct photosynthetic measurements, targeted metabolite quantification, lignin measurement, and functional validation.

## Data Availability

The RNA-seq raw reads generated in this study have been deposited in the NCBI Sequence Read Archive (SRA) under BioProject accession PRJNA1481826. The metabolome raw data have been deposited in the OMIX database of the China National Center for Bioinformation (https://ngdc.cncb.ac.cn/omix/) under accession OMIX018860. The processed transcriptome data and other supporting datasets are provided in the article and Supplementary Materials. Further inquiries can be directed to the corresponding author.

## Supplementary Materials

The following supporting information can be downloaded at: https://www.mdpi.com/article/10.3390/ijms27167424/s1.

## Abbreviations

The following abbreviations are used in this manuscript:

AAT	Aspartate aminotransferase
ANOVA	Analysis of variance
BP	Biological process
C	Cold stress (4 °C) without selenium
C+10, C+20, C+40	Cold stress (4 °C) with a foliar spray of 10, 20, or 40 mg L^−1^ Na_2_SeO_3_, respectively
CAD	Cinnamyl alcohol dehydrogenase
CAT	Catalase
CC	Cellular component
cDNA	Complementary DNA
CK	Control check (normal growth, without cold stress or selenium)
CSE	Caffeoylshikimate esterase
CSe	Cold stress supplemented with selenium (20 mg L^−1^ Na_2_SeO_3_); group used for the omics analysis
DAMs	Differentially accumulated metabolites
DEGs	Differentially expressed genes
FC	Fold change
FLS	Flavonol synthase
GO	Gene Ontology
H_2_O_2_	Hydrogen peroxide
KEGG	Kyoto Encyclopedia of Genes and Genomes
LC-MS	Liquid chromatography–mass spectrometry
LDH	Lactate dehydrogenase
MDA	Malondialdehyde
MF	Molecular function
mRNA	Messenger RNA
Na_2_SeO_3_	Sodium selenite
NBT	Nitro-blue tetrazolium
NCBI	National Center for Biotechnology Information
OASTL	O-acetylserine(thiol)lyase
PCA	Principal component analysis
*PER*	Peroxidase (gene)
PGDH	Phosphoglycerate dehydrogenase
POD	Peroxidase (enzyme activity)
PPFD	Photosynthetic photon flux density
QC	Quality control
qRT-PCR	Quantitative real-time polymerase chain reaction
RNA-seq	RNA sequencing
ROS	Reactive oxygen species
SAHH	S-adenosylhomocysteine hydrolase
SAM	S-adenosylmethionine
SAT	Serine acetyltransferase
SD	Standard deviation
Se	Selenium
SOD	Superoxide dismutase
SRA	Sequence Read Archive
TBA	Thiobarbituric acid
TCA	Trichloroacetic acid
UGT	UDP-glycosyltransferase
UHPLC	Ultra-high-performance liquid chromatography
VIP	Variable importance in projection
