# Supplementary material for: Integrated Transcriptomic and Metabolomic Analysis Reveals Molecular Responses Associated with Foliar Selenium Treatment Under Cold Stress in Rapeseed (Brassica napus L.) Seedlings
Source: Int J Mol Sci. 2026 Aug 19;27(16):7424. doi: 10.3390/ijms27167424 (PMC13513434; doi:10.3390/ijms27167424)
Supplement: Supplementary file 1 [file ijms-27-07424-s001.zip › Figure S1.pdf]

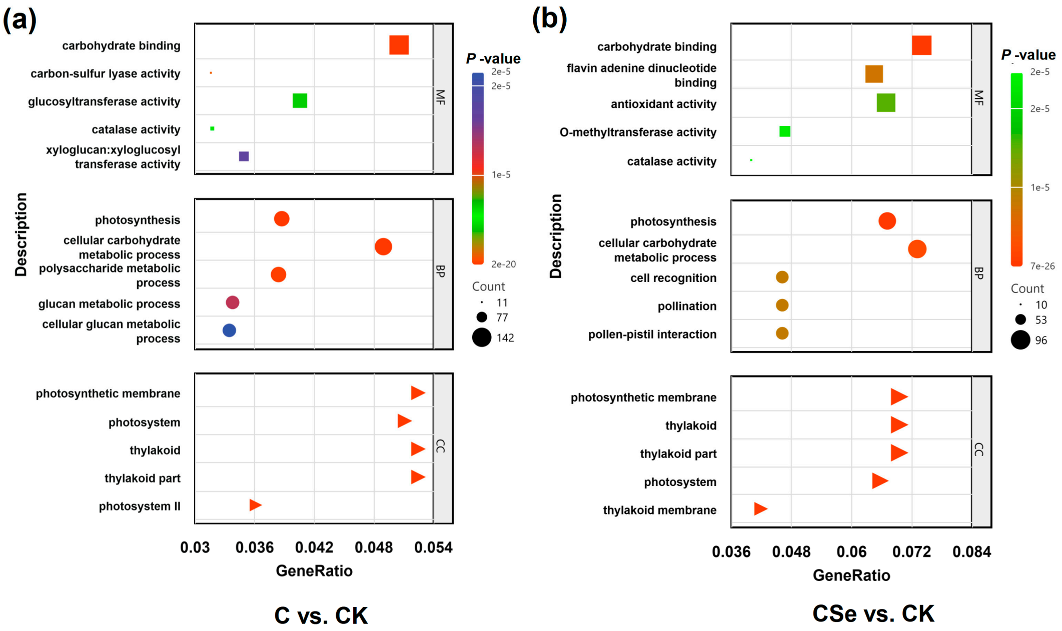

Figure S1. GO enrichment of the DEGs in the C vs. CK and CSe vs. CK comparisons. (**a**) GO enrichment in the C vs. CK comparison. (**b**) GO enrichment in the CSe vs. CK comparison. The terms are divided into the molecular function (MF), biological process (BP) and cellular component (CC) categories. The x-axis represents the GeneRatio, the dot/symbol size indicates the number of DEGs, and symbol color represents the BH-adjusted *P*-value. CK, well-watered control; C, 4 °C cold stress; CSe, cold stress supplemented with selenium.
