# Supplementary material for: Integrated Transcriptomic and Metabolomic Analysis Reveals Molecular Responses Associated with Foliar Selenium Treatment Under Cold Stress in Rapeseed (Brassica napus L.) Seedlings
Source: Int J Mol Sci. 2026 Aug 19;27(16):7424. doi: 10.3390/ijms27167424 (PMC13513434; doi:10.3390/ijms27167424)
Supplement: Supplementary file 1 [file ijms-27-07424-s001.zip › Figure S3.pdf]

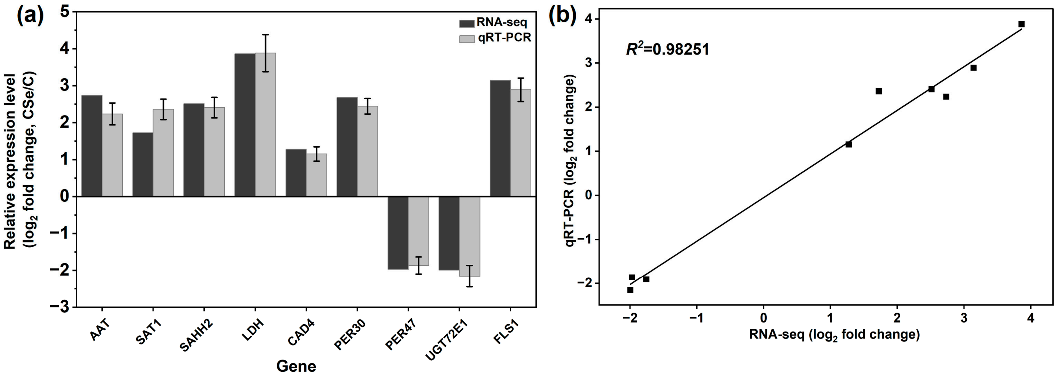

Figure S3. qRT-PCR confirmation of selected RNA-seq expression patterns. Nine DEGs from the three key pathways were selected. (**a**) Relative expression of the selected genes measured by qRT-PCR and by RNA-seq. The genes were *AAT, SAT1, SAHH2, LDH* (cysteine and methionine metabolism); *CAD4, PER30, PER47, UGT72E1* (phenylpropanoid biosynthesis); and *FLS1* (flavonoid biosynthesis). Values are the log2 fold change of the CSe group relative to the C group. The same RNA samples were used for qRT-PCR and RNA-seq. Relative expression was calculated with the $2^{-\Delta\Delta CT}$ method, with the Brassica napus L. actin gene as the internal reference. Data are means $\pm$ SD of three biological replicates, each with three technical replicates. (**b**) Correlation between the RNA-seq and qRT-PCR data ($R^2 = 0.98$). The qRT-PCR results agreed with the RNA-seq data, confirming the reliability of the transcriptome.
