# Supplementary material for: Integrated Transcriptomic and Metabolomic Analysis Reveals Molecular Responses Associated with Foliar Selenium Treatment Under Cold Stress in Rapeseed (Brassica napus L.) Seedlings
Source: Int J Mol Sci. 2026 Aug 19;27(16):7424. doi: 10.3390/ijms27167424 (PMC13513434; doi:10.3390/ijms27167424)
Supplement: Supplementary file 1 [file ijms-27-07424-s001.zip › Figure S4.pdf]

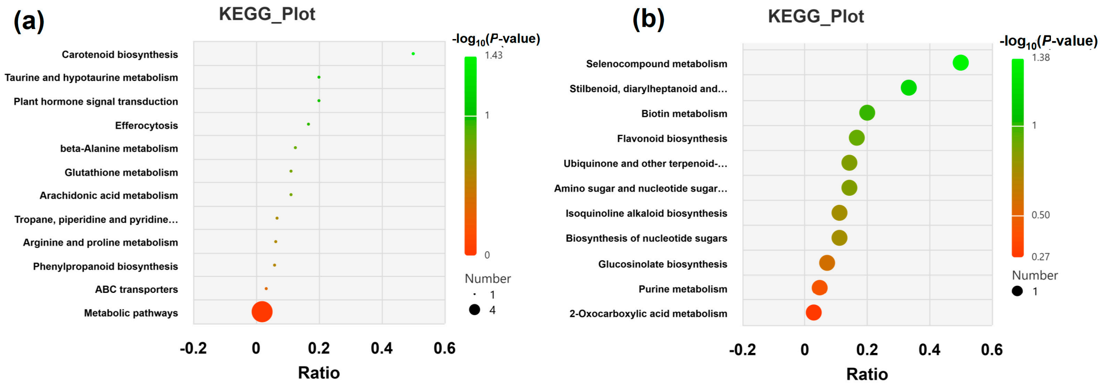

Figure S4. Kyoto Encyclopedia of Genes and Genomes (KEGG) pathway enrichment of the differentially accumulated metabolites (DAMs) in the (**a**) C vs. CK comparison and (**b**) CSe vs. CK comparison. In both panels, the x-axis represents the Ratio (the proportion of DAMs annotated to each pathway), the dot size indicates the number of DAMs, and the color represents the significance level (-$\log_{10}$ *P*-value). CK, well-watered control; C, 4 °C cold stress; CSe, cold stress supplemented with selenium.
