# Supplementary material for: Integrated Transcriptomic and Metabolomic Analysis Reveals Molecular Responses Associated with Foliar Selenium Treatment Under Cold Stress in Rapeseed (Brassica napus L.) Seedlings
Source: Int J Mol Sci. 2026 Aug 19;27(16):7424. doi: 10.3390/ijms27167424 (PMC13513434; doi:10.3390/ijms27167424)
Supplement: Supplementary file 1 [file ijms-27-07424-s001.zip › Figure S5.pdf]

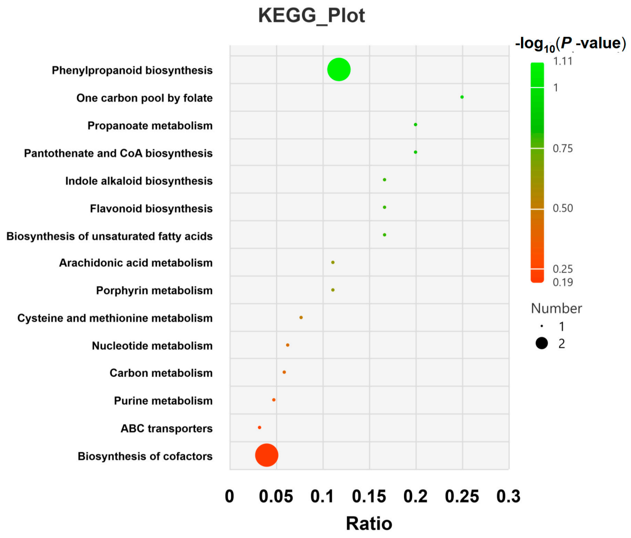

Figure S5. Kyoto Encyclopedia of Genes and Genomes (KEGG) pathway enrichment of the differentially accumulated metabolites (DAMs) in the CSe vs. C comparison. The x-axis represents the Ratio, symbol size indicates the number of candidate DAMs assigned to each pathway, and symbol color represents $-\log_{10}$ of the nominal *P*-value. Phenylpropanoid biosynthesis showed the highest nominal enrichment among the displayed candidate pathways. C, 4 °C cold stress; CSe, cold stress supplemented with selenium.
