# Supplementary material for: Integrated Transcriptomic and Metabolomic Analysis Reveals Molecular Responses Associated with Foliar Selenium Treatment Under Cold Stress in Rapeseed (Brassica napus L.) Seedlings
Source: Int J Mol Sci. 2026 Aug 19;27(16):7424. doi: 10.3390/ijms27167424 (PMC13513434; doi:10.3390/ijms27167424)
Supplement: Supplementary file 1 [file ijms-27-07424-s001.zip › Figure S6.pdf]

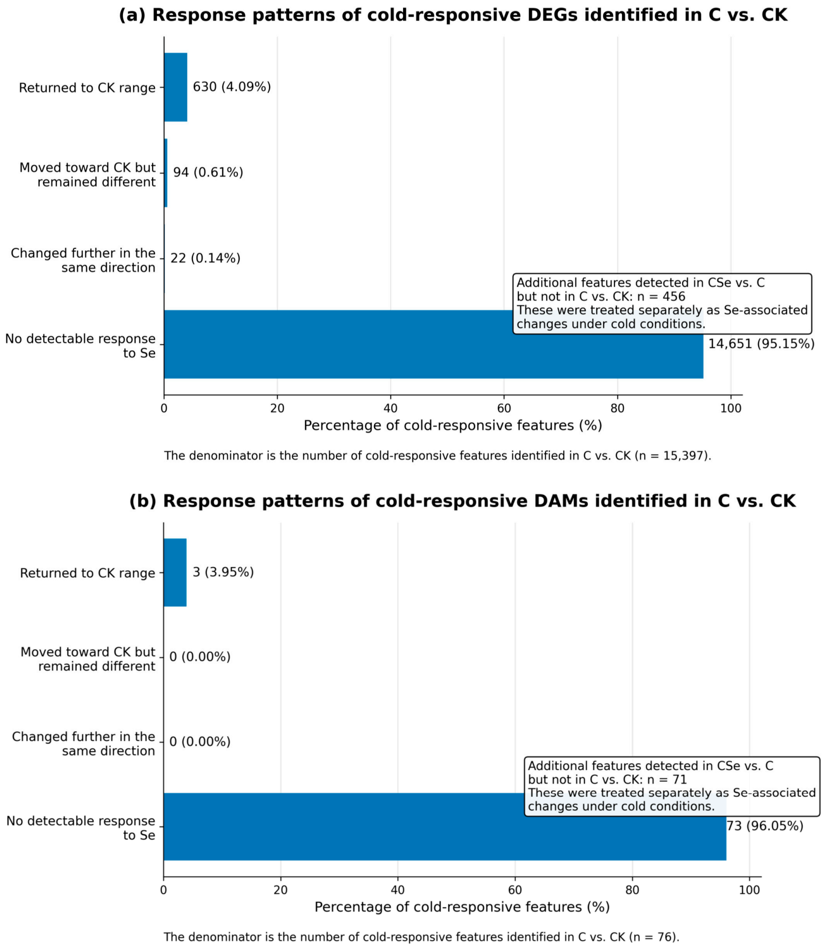

Figure S6. Figure S6. Classification of cold responsive transcriptomic and metabolomic features across CK, C, and CSe. (**a**) Cold responsive DEGs identified in C vs. CK were classified according to their subsequent behavior in CSe. Among 15,397 cold responsive genes, 630 returned to the CK range, 94 moved toward CK but remained different, 22 changed further in the same direction, and 14,651 showed no detectable response to Se. An additional 456 genes were detected in CSe vs. C but not in C vs. CK and were therefore treated separately as Se associated changes under cold conditions. (**b**) Cold responsive DAMs identified in C vs. CK were classified in the same way. Among 76 cold responsive metabolites, 3 returned to the CK range and 73 showed no detectable response to Se. No metabolites were assigned to the categories moved toward CK but remained different or changed further in the same direction. An additional 71 metabolites were detected in CSe vs. C but not in C vs. CK and were therefore treated separately as Se associated changes under cold conditions. CK, normal temperature control. C, cold stress. CSe, cold stress with Se treatment.
