# Supplementary material for: Integrated Transcriptomic and Metabolomic Analysis Reveals Molecular Responses Associated with Foliar Selenium Treatment Under Cold Stress in Rapeseed (Brassica napus L.) Seedlings
Source: Int J Mol Sci. 2026 Aug 19;27(16):7424. doi: 10.3390/ijms27167424 (PMC13513434; doi:10.3390/ijms27167424)
Supplement: Supplementary file 1 [file ijms-27-07424-s001.zip › Figure S7.pdf]

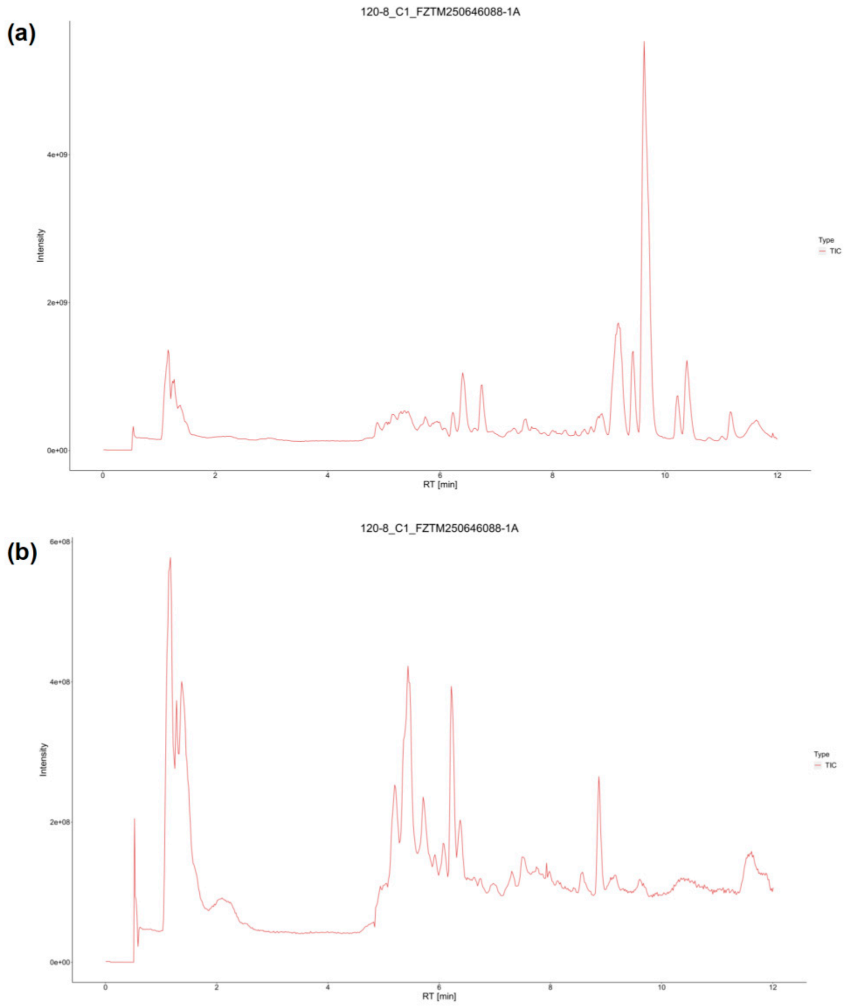


**Figure S7.** Representative total ion chromatograms of the pooled quality-control samples acquired in (**a**) positive and (**b**) negative electrospray ionization modes. The pooled QC sample was prepared by mixing equal volumes of all biological samples and was used to monitor the stability of the LC–MS system.
