# Supplementary material for: Integrated Transcriptomic and Metabolomic Analysis Reveals Molecular Responses Associated with Foliar Selenium Treatment Under Cold Stress in Rapeseed (Brassica napus L.) Seedlings
Source: Int J Mol Sci. 2026 Aug 19;27(16):7424. doi: 10.3390/ijms27167424 (PMC13513434; doi:10.3390/ijms27167424)
Supplement: Supplementary file 1 [file ijms-27-07424-s001.zip › Table S1.pdf]

Table S1. The statistic of RNA-Seq data of the 9 samples.

Samples	Clean reads	Total Clean Bases (Gb)	Mapped reads (%)	GC Content (%)	Q30 (%)
CK-1	46,896,476	7.03	46,502,785(99.16%)	46.85%	98.88%
CK-2	46,980,152	7.05	46,576,596(99.14%)	46.69%	98.78%
CK-3	45,388,314	6.81	42,838,259(94.38%)	46.52%	98.89%
C-1	46,035,012	6.91	45,171,274(98.12%)	46.81%	97.7%
C-2	45,772,122	6.87	45,154,252(98.65%)	47.42%	98.96%
C-3	45,203,412	6.78	44,598,371(98.66%)	46.74%	98.97%
CSe-1	44,750,130	6.71	44,260,059(98.9%)	47.03%	99%
CSe-2	45,118,238	6.77	44,238,196(98.05%)	46.66%	97.89%
CSe-3	53,847,472	8.08	52,823,250(98.1%)	46.91%	97.72%
