# Supplementary material for: Integrated Transcriptomic and Metabolomic Analysis Reveals Molecular Responses Associated with Foliar Selenium Treatment Under Cold Stress in Rapeseed (Brassica napus L.) Seedlings
Source: Int J Mol Sci. 2026 Aug 19;27(16):7424. doi: 10.3390/ijms27167424 (PMC13513434; doi:10.3390/ijms27167424)
Supplement: Supplementary file 1 [file ijms-27-07424-s001.zip › Table S4.pdf]

Table S4. Significant gene–metabolite correlations (edges) of the exploratory correlation network in the three key pathways (Pearson |*r*| ≥ 0.8, *P* < 0.05; n = 6 samples).

Gene (node)	Gene ID (ZS11)	Gene pathway	Metabolite (node)	Metabolite KEGG ID	Metabolite pathway	Pearson r	P-value	Correlation
AAT(cyt)	ZS11A09G012100	Cysteine and methionine metabolism	3-Sulfopyruvic acid	C05528	Cysteine and methionine metabolism	0.991	0.0001	Positive
CSE	ZS11A04G032000	Phenylpropanoid biosynthesis	3-Sulfopyruvic acid	C05528	Cysteine and methionine metabolism	0.99	0.0001	Positive
SAT1	ZS11A08G000240	Cysteine and methionine metabolism	3-Sulfopyruvic acid	C05528	Cysteine and methionine metabolism	0.947	0.0042	Positive
FLS1	ZS11C09G008720	Flavonoid biosynthesis	3-Sulfopyruvic acid	C05528	Cysteine and methionine metabolism	0.94	0.0054	Positive
SAHH2#1	ZS11C01G040740	Cysteine and methionine metabolism	3-Sulfopyruvic acid	C05528	Cysteine and methionine metabolism	0.93	0.0073	Positive
PER30	ZS11C01G043680	Phenylpropanoid biosynthesis	3-Sulfopyruvic acid	C05528	Cysteine and methionine metabolism	0.905	0.0132	Positive
UGT72E1	ZS11A03G042910	Phenylpropanoid biosynthesis	3-Sulfopyruvic acid	C05528	Cysteine and methionine metabolism	-0.887	0.0183	Negative
AAT(chl)	ZS11C07G060030	Cysteine and	3-Sulfopyruvic	C05528	Cysteine and	0.883	0.0197	Positive

		methionine metabolism	acid		methionine metabolism			
SAHH2#2	ZS11A01G031230	Cysteine and methionine metabolism	3-Sulfopyruvic acid	C05528	Cysteine and methionine metabolism	0.874	0.0227	Positive
PER34	ZS11A01G023950	Phenylpropanoid biosynthesis	3-Sulfopyruvic acid	C05528	Cysteine and methionine metabolism	-0.832	0.0398	Negative
LDH	ZS11A01G019000	Cysteine and methionine metabolism	3-Sulfopyruvic acid	C05528	Cysteine and methionine metabolism	0.828	0.0417	Positive
PER30	ZS11C01G043680	Phenylpropanoid biosynthesis	Coniferaldehyde	C02666	Phenylpropanoid biosynthesis	0.975	0.0009	Positive
SAHH2#1	ZS11C01G040740	Cysteine and methionine metabolism	Coniferaldehyde	C02666	Phenylpropanoid biosynthesis	0.969	0.0014	Positive
SAHH2#2	ZS11A01G031230	Cysteine and methionine metabolism	Coniferaldehyde	C02666	Phenylpropanoid biosynthesis	0.959	0.0025	Positive
LDH	ZS11A01G019000	Cysteine and methionine metabolism	Coniferaldehyde	C02666	Phenylpropanoid biosynthesis	0.957	0.0027	Positive
AAT(chl)	ZS11C07G060030	Cysteine and methionine metabolism	Coniferaldehyde	C02666	Phenylpropanoid biosynthesis	0.951	0.0036	Positive
UGT72E1	ZS11A03G042910	Phenylpropanoid	Coniferaldehyde	C02666	Phenylpropanoid	-0.935	0.0062	Negative

		biosynthesis			biosynthesis			
AAT(cyt)	ZS11A09G012100	Cysteine and methionine metabolism	Coniferaldehyde	C02666	Phenylpropanoid biosynthesis	0.885	0.019	Positive
CSE	ZS11A04G032000	Phenylpropanoid biosynthesis	Coniferaldehyde	C02666	Phenylpropanoid biosynthesis	0.87	0.0242	Positive
PER47#1	ZS11A03G055710	Phenylpropanoid biosynthesis	Coniferaldehyde	C02666	Phenylpropanoid biosynthesis	-0.825	0.0434	Negative
PER34	ZS11A01G023950	Phenylpropanoid biosynthesis	Homoeriodictyol	C09756	Flavonoid biosynthesis	0.963	0.002	Positive
CAD4	ZS11C05G047170	Phenylpropanoid biosynthesis	Homoeriodictyol	C09756	Flavonoid biosynthesis	-0.963	0.0021	Negative
UGT72E1	ZS11A03G042910	Phenylpropanoid biosynthesis	Homoeriodictyol	C09756	Flavonoid biosynthesis	0.924	0.0084	Positive
PER25	ZS11C04G069970	Phenylpropanoid biosynthesis	Homoeriodictyol	C09756	Flavonoid biosynthesis	0.872	0.0237	Positive
SAHH2#2	ZS11A01G031230	Cysteine and methionine metabolism	Homoeriodictyol	C09756	Flavonoid biosynthesis	-0.867	0.0254	Negative
AAT(cyt)	ZS11A09G012100	Cysteine and methionine metabolism	Homoeriodictyol	C09756	Flavonoid biosynthesis	-0.858	0.0286	Negative
PER30	ZS11C01G043680	Phenylpropanoid biosynthesis	Homoeriodictyol	C09756	Flavonoid biosynthesis	-0.852	0.0314	Negative
SAHH2#1	ZS11C01G040740	Cysteine and methionine	Homoeriodictyol	C09756	Flavonoid biosynthesis	-0.835	0.0388	Negative

		metabolism						
LDH	ZS11A01G019000	Cysteine and methionine metabolism	Homoeriodictyol	C09756	Flavonoid biosynthesis	-0.835	0.0386	Negative
PER47#1	ZS11A03G055710	Phenylpropanoid biosynthesis	Homoeriodictyol	C09756	Flavonoid biosynthesis	0.828	0.0418	Positive
AAT(chl)	ZS11C07G060030	Cysteine and methionine metabolism	Homoeriodictyol	C09756	Flavonoid biosynthesis	-0.814	0.0486	Negative
