# Supplementary material for: Integrated Transcriptomic and Metabolomic Analysis Reveals Molecular Responses Associated with Foliar Selenium Treatment Under Cold Stress in Rapeseed (Brassica napus L.) Seedlings
Source: Int J Mol Sci. 2026 Aug 19;27(16):7424. doi: 10.3390/ijms27167424 (PMC13513434; doi:10.3390/ijms27167424)
Supplement: Supplementary file 1 [file ijms-27-07424-s001.zip › Table S5.pdf]

Table S5. The primers of 9 genes for qRT-PCR validation.

Gene	Transcript_id	Primer name (Sense/Anti-sense primer)	Nucleotide sequence
AAT	ZS11A09G012100	F	5' GAACCAACACCCTCAAACGC 3'
		R	5' GCGTTTGAAGCAGTGAGGTG 3'
SAT1	ZS11A08G000240	F	5' TTGGGACCTGTGGCAGTTAC 3'
		R	5' CATGTGCCTGTCCAGTTTGC 3'
SAHH2	ZS11C01G040740	F	5' GTTTAGCCCCAACACGCTTG 3'
		R	5' GAGATAACCAGAGTGCCCCG 3'
LDH	ZS11A01G019000	F	5' TCACGGGACAAGTCATGACG 3'
		R	5' GTGCTGTGTTTAGCTCGCAG 3'
CAD4	ZS11C05G047170	F	5' CAAGGGAGGTAACGAGCAGG 3'
		R	5' CTTCCTCTTGCGTGTGTTGC 3'
PER30	ZS11C01G043680	F	5' ACGGGAACAACATAGGCCTG 3'
		R	5' AACTCGTTCGTATCCACCGG 3'
PER47	ZS11A03G055710	F	5' CATCCTCTTCGCCTACGGTG 3'
		R	5' CACAAGGACCTACGCAACGA 3'
UGT72E1	ZS11A03G042910	F	5' CTCCGCTCCTCCTTACCTCT 3'
		R	5' CAAACAAGCCGGAAGAGCAC 3'
FLS1	ZS11C09G008720	F	5' GATGCTGTCGGAGATTGGCT 3'
		R	5' CTGGTTCGAACTCCATGTCCA 3'
Actin	Actin (reference)	F	5' TGGGTTTGCTGGTGACGAT 3'
		R	5' TGCCTAGGACGACCAACAATACT 3'
